# Methyltransferase METTL1 regulates MSC mRNA stability via m7G modification in acute pancreatitis

**DOI:** 10.1038/s41419-026-09002-7

**Published:** 2026-06-23

**Authors:** Xufeng Tao, Xiaonan Zhang, Fangyue Guo, Yunshu Zhang, Lei Zhang, Han Yu, Deshi Dong, Dong Shang, Hong Xiang

**Affiliations:** 1 Department of Pharmacy, First Affiliated Hospital of Dalian Medical University, Dalian, China; 2 Laboratory of Integrative Medicine, First Affiliated Hospital of Dalian Medical University, Dalian, China; 3 Institute (College) of Integrative Medicine, Dalian Medical University, Dalian, China; 4 Department of Pharmacy, Dalian Medical University, Dalian, China; 5 Department of General Surgery, First Affiliated Hospital of Dalian Medical University, Dalian, China

**Keywords:** Acute pancreatitis, Acute inflammation

## Abstract

Acute pancreatitis (AP) is a serious inflammatory disease with significant morbidity, yet its underlying molecular mechanisms remain incompletely understood. This study reveals a novel epitranscriptomic pathway in AP pathogenesis centered on METTL1-mediated N7-methylguanosine (m7G) RNA modification. We found that METTL1 expression and global m7G levels were significantly elevated in serum from AP patients, pancreatic tissues of sodium taurocholate-induced AP mice, and in vitro models of LPS-polarized macrophages and STC-injured pancreatic acinar cells. Through integrated multi-omics analysis combining m7G methylome mapping and transcriptome profiling, we identified Musculin (MSC) as a key target whose mRNA stability is enhanced by METTL1-mediated m7G modification. Functional experiments demonstrated that MSC upregulation activates TNF signaling through phosphorylation of NF-κB, JNK, and MAPK proteins, thereby promoting macrophage M1 polarization and pancreatic acinar cell injury. The pathological significance of this pathway was confirmed in vivo, where pancreas-targeted knockdown of Mettl1 significantly attenuated AP severity. Furthermore, mechanistic studies using a catalytic-dead METTL1 mutant established that both the methyltransferase activity of METTL1 and subsequent TNF signaling activation are essential for driving inflammatory responses. Our findings delineate a previously unrecognized METTL1-m7G-MSC-TNF signaling axis that promotes AP progression, highlighting the therapeutic potential of targeting METTL1-mediated epitranscriptomic modification in inflammatory diseases.

## Introduction

Acute pancreatitis (AP) is the most common abdominal emergencies worldwide, with increasing incidence rates in all age groups. Approximately 20% of AP patients progress to severe cases, characterized by pancreatic necrosis and/or organ failure, with a mortality rate of up to 20% [1, 2]. Of particular noteworthy are the high risk of AP recurrence and progression to chronic pancreatitis, as well as the unexpectedly high incidence of post-AP diabetes and exocrine insufficiency. Despite these challenges, the complex molecular network underlying AP pathogenesis remains poorly understood, leaving current clinical strategies limited to supportive care.

Pancreatic acinar cells (PACs) are the primary cell population undergo damage through overactivation of pancreatic zymogens, considered as the initiating event in AP pathogenesis [3]. The subsequent release of damage-associated molecular patterns (DAMPs) from injured PACs aggravates pancreatic inflammation by recruiting immune cells to establish a proinflammatory microenvironment. Among these immune responders, macrophages are the most abundant population during early AP stages, primarily presenting the proinflammatory M1 phenotype [4, 5]. The crosstalk between damaged PACs and M1-like macrophages triggers intense local and/or systemic inflammatory cascades [6, 7]. Therefore, these cellular pathological events are recognized promising therapeutic targets for AP intervention.

Epitranscriptomic modifications are increasingly recognized as key regulators of physiological processes and disease progression, and RNA chemical modification constitutes a central layer of this regulation. The essential role of RNA modification in PACs injury during AP and macrophage polarization in PDAC has been fully validated [8, 9]. With more than 160 naturally occurring RNA modifications identified to date, these marks collectively shape RNA processing, stability and translation, thereby controlling cell-state transitions under homeostatic and pathological conditions [10, 11]. Among various RNA modifications, N7-methylguanosine (m7G) has recently attracted significant scientific interest due to it is classically known as a canonical 5′ cap structure, yet accumulating evidence indicates that internal m7G modifications also exist across RNA species (including tRNA and mRNA) and participate in gene regulatory programs [12–14]. In mammals, methyltransferase-like 1 (METTL1) serves as the most well-studied regulator of m7G, partnering with its corresponding cofactor WD repeat domain 4 (WDR4) to catalyze m7G installation in tRNA, miRNA, and mRNA. Increasing evidence indicates aberrant m7G patterns have been extensively documented in oncogenesis [15], but their potential roles in inflammatory pathologies, particularly AP, remain largely unexplored.

In this study, we focused on the function of METTL1-mediated m7G mRNA modification in macrophage M1 polarization and PACs injury during early AP stages. Our findings provide novel insights into the epitranscriptomic regulation of AP progression and suggest potential therapeutic strategies targeting m7G modification.

## Results

### METTL1-mediated m7G methylation was up-regulated in AP pathogenesis

**Fig. 1:**
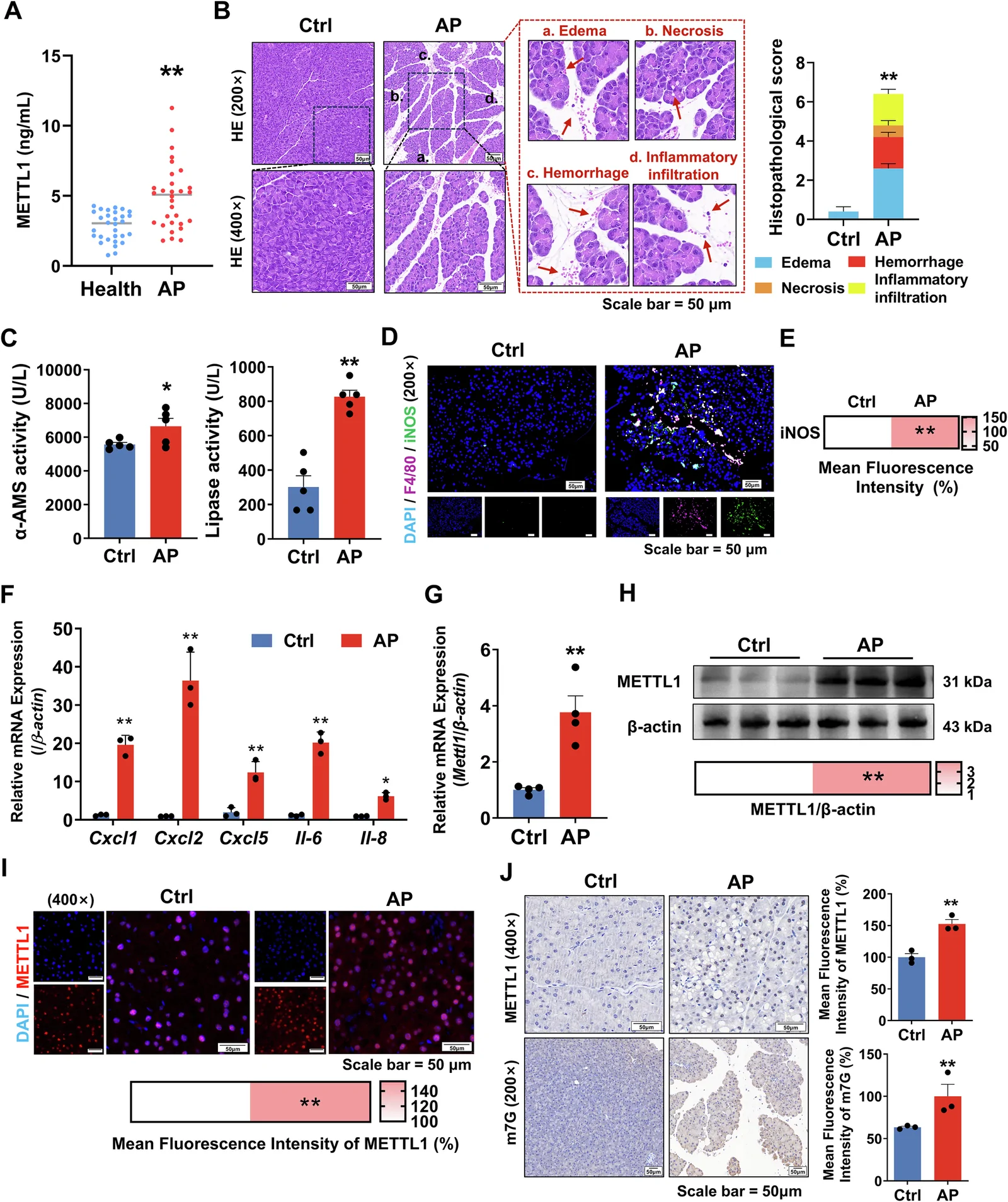
METTL1-mediated m7G methylation in AP pathogenesis. **A** The serum METTL1 levels in healthy individuals and AP patients (*n* = 35). Data are presented as mean ± SEM; **P < 0.01 vs. Health. **B** Representative H&E staining of pancreatic sections from Ctrl and STC-induced AP mice (200× and 400×), with representative images highlighting edema, necrosis, hemorrhage, and inflammatory infiltration (arrows), and histopathological scoring. **C** The serum α-amylase activity and lipase levels in Ctrl and AP mice. **D** Immunofluorescence staining of F4/80 (magenta) and iNOS (green) with DAPI (blue) in pancreatic sections (200×). **E** Quantification of mean fluorescence intensity for iNOS. **F** qPCR analysis of inflammatory chemokines/cytokines (*Cxcl1, Cxcl2, Cxcl5, Il-6, Il-8*) in mouse pancreatic tissues. **G** qPCR analysis of *Mettl1* expression in mouse pancreatic tissues. **H** Western blot analysis of METTL1 protein in pancreatic tissues with β-actin as loading control and densitometric quantification (METTL1/β-actin). **I** Immunofluorescence staining of METTL1 (red) with DAPI (blue) in pancreatic sections (400×) and quantification of METTL1 mean fluorescence intensity. **J** Immunohistochemical staining of METTL1 (400×) and m7G (200×) in pancreatic sections with semi-quantitative analysis. Data are presented as mean ± SEM; *P < 0.05, **P < 0.01 vs. Ctrl.

We firstly observed the serum METTL1 concentration of AP patients was significantly higher than that of healthy individuals, suggesting that METTL1 as a potential biomarker and its functions during AP needs further exploration (Fig. 1A). At the animal level, intact acinar architectures were observed in Ctrl mice, while STC-induced AP mice displayed typical pancreatic injury featured by interstitial edema, acinar necrosis, hemorrhage, and prominent inflammatory cell infiltration, with a significantly increased histopathological score (Fig. 1B). Consistently, serum α-amylase and lipase activities were higher in AP mice than in Ctrl mice (Fig. 1C**)**. In pancreatic sections of AP mice, the number of iNOS⁺ cells are increased (Fig. 1D, E), and at the same time, proinflammatory chemokines/cytokines in pancreatic tissues (including *Cxcl1*, *Cxcl2*, *Cxcl5*, *Il-6*, and *Il-8*) are significantly elevated (Fig. 1F), which indicates an increased infiltration of M1-like macrophages. At both the gene (Fig. 1G) and protein levels (Fig. 1H–J), compared to the Ctrl, METTL1 expression was significantly increased in pancreatic tissue of AP mice, with the enhanced global m7G positive area (brown area) (Fig. 1J). These results indicate that METTL1-mediated m7G methylation is elevated during AP and may be associated with pancreatic injury and pro-inflammatory macrophage activation.

### METTL1-mediated m7G methylation drives macrophage M1 polarization

**Fig. 2:**
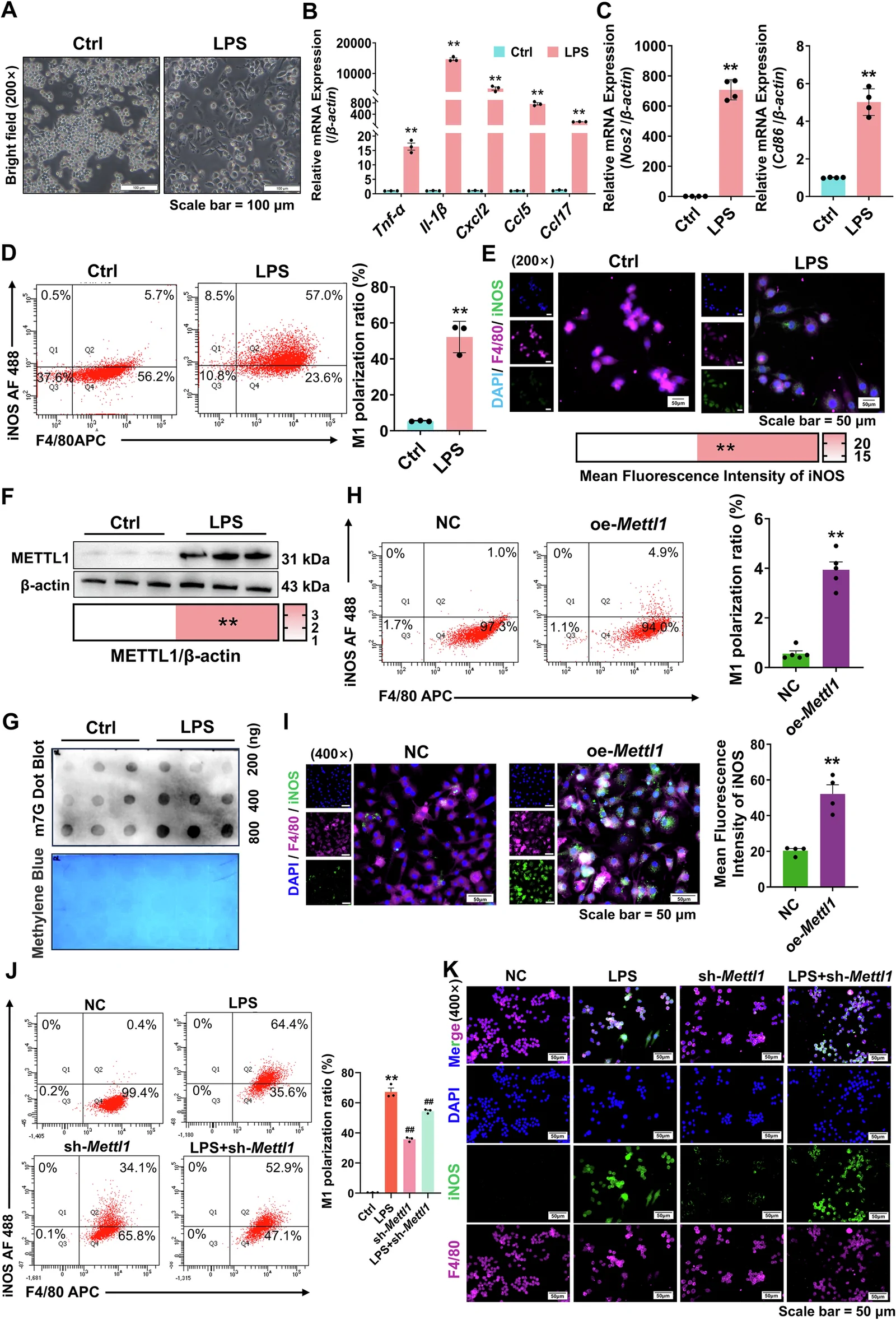
METTL1-mediated m7G methylation drives macrophage M1 polarization. **A** Representative bright-field images of macrophages with or without LPS stimulation (200×). **B** qPCR analysis of inflammatory factor mRNA levels (*Tnf-α, Il-1β, Cxcl2, Ccl5, Ccl17*) in Ctrl and LPS-treated macrophages (*n* = 4). **C** qPCR analysis of M1-associated markers *Nos2* and *Cd86* (*n* = 4). **D** Flow cytometry analysis of F4/80 and iNOS expression in Ctrl and LPS groups, and quantification of M1 polarization ratio (F4/80⁺iNOS⁺) (*n* = 3). **E** Immunofluorescence staining of F4/80 (magenta) and iNOS (green) with DAPI (blue) in Ctrl and LPS-treated macrophages (200×), and quantification of iNOS mean fluorescence intensity. **F** Western blot analysis of METTL1 protein levels in macrophages with β-actin as loading control. **G** m7G dot blot analysis of global m7G levels in total RNA from Ctrl and LPS-treated macrophages (200–800 ng), with methylene blue staining as loading control. **H** Flow cytometry analysis and quantification of M1 polarization ratio (F4/80⁺iNOS⁺) in NC and oe-*Mettl1* groups (*n* = 5). **I** Immunofluorescence staining of F4/80 (magenta) and iNOS (green) with DAPI (blue) in NC and oe-*Mettl1* groups (400×), and quantification of iNOS mean fluorescence intensity. **J** Flow cytometry analysis of F4/80 and iNOS expression in LPS, sh-*Mettl1* and LPS+sh-*Mettl1* groups, and quantification of M1 polarization ratio (F4/80⁺iNOS⁺). **K** Immunofluorescence analysis of macrophage inflammatory activation following *Mettl1* knockdown, iNOS (green) and F4/80 (magenta), with nuclei counterstained by DAPI (blue). Data are presented as mean ± SEM; **P < 0.01 vs. NC, ^##^*P* < 0.01 vs. LPS.

To evaluate whether METTL1-mediated m7G methylation contributes to macrophage M1 polarization, we stimulated mouse macrophages RAW264.7 cells with LPS. Compared with the Ctrl, LPS-treated macrophages exhibited a more spread and irregular morphology, accompany by the markedly increased mRNA levels of inflammatory mediators, including *Tnf-α, Il-1β, Cxcl2, Ccl5, and Ccl17*, as well as elevated expression of M1-associated markers *Nos2* (iNOS) and *Cd86* (Fig. 2A–C). Flow cytometry further confirmed an increased proportion of F4/80⁺ iNOS⁺ macrophages after LPS stimulation, indicating resting macrophages transform into M1 phenotype, which is also confirmed by immunofluorescence (Fig. 2D, E). Moreover, METTL1 protein expression and global m7G modification were both elevated in LPS-treated macrophages by western blotting and m7G dot blot analysis (Fig. 2F, G). To further verify METTL1’s function, we overexpressed *Mettl1* in macrophages, and found that increased expression of *Mettl1* was responsible for the increased fraction of F4/80⁺ iNOS⁺ macrophages (Fig. 2H, I). However, knockdown of Mettl1 in vitro reduced the level of LPS-induced M1 polarization in macrophages (Fig. 2J, K). These results indicate that METTL1 promotes macrophage M1 polarization in vitro by increasing global m7G methylation.

### *Mettl1* knockdown alleviates pancreatic injury and inflammatory responses induced by AP

**Fig. 3:**
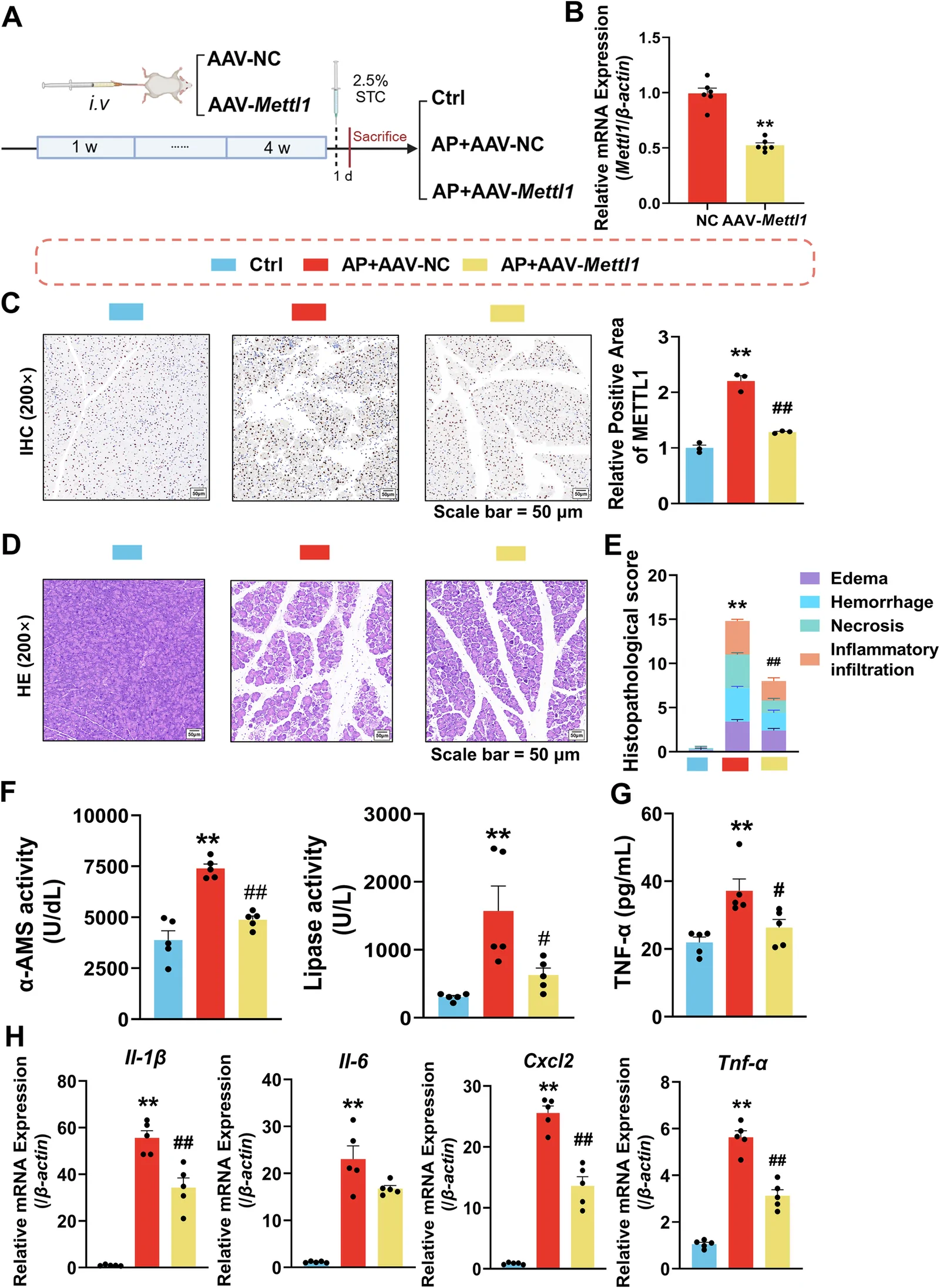
*Mettl1* knockdown alleviates AP-induced PACs injury and inflammatory cascades in vivo. **A** Schematic of the experimental design. Mice received intravenous injection of AAV-NC or AAV-*Mettl1*, followed by induction of AP with STC for 24 h. **B** qPCR validation of pancreatic *Mettl1* knockdown efficiency in AP mice. **C** Immunohistochemical staining of METTL1 in pancreatic sections (200×) and quantification of METTL1-positive area. **D** Representative H&E staining of pancreatic sections from Ctrl, AP + AAV-NC, and AP + AAV-*Mettl1* mice (200×). **E** Quantification of histopathological scores, including edema, hemorrhage, necrosis, and inflammatory infiltration. **F** The serum α-amylase activity and lipase levels. **G** The serum TNF-α levels measured by ELISA. **H** qPCR analysis of inflammatory mediators in pancreatic tissues *Il-1β, Il-6, Tnf-α, Ccl5* and *Cxcl2*. Data are presented as mean ± SEM. **P < 0.01 vs. NC, ^#^*P* < 0.05, ^##^*P* < 0.01 vs. AP + AVV-NC.

To further determine the functional contribution of METTL1 in vivo, mice were intravenously injected with AAV-NC or AAV-*Mettl1* to achieve pancreas-targeted *Mettl1* knockdown, followed by induction of AP (Fig. 3A). qPCR confirmed that *Mettl1* expression was significantly reduced in the pancreas of AP mice receiving AAV-*Mettl1* compared with AAV-NC (Fig. 3B). Consistently, immunohistochemical staining showed robust METTL1 upregulation in AP + AAV-NC pancreatic tissues, whereas AAV-*Mettl1* markedly decreased the METTL1-positive area (Fig. 3C). H&E staining revealed typical AP lesions in AP + AAV-NC mice, including acinar necrosis, edema, and inflammatory infiltration with an increased histopathological score, which were substantially attenuated by AAV-*Mettl1* (Fig. 3D, E). In parallel, serum α-amylase and lipase activities were markedly elevated in AP + AAV-NC mice, but decreased in the AP + AAV-*Mettl1* group (Fig. 3F). Furthermore, increased serum TNF-α levels in AP mice were reduced upon *Mettl1* knockdown (Fig. 3G). In addition, AAV-*Mettl1* significantly blunted the AP-associated induction of *Il-1β, Il-6, Cxcl2, Ccl5* and *Tnf-α* (Fig. 3H). These results indicate that Mettl1 contributes to AP injury and inflammatory responses in vivo.

### METTL1 catalytic activity is required for m7G-dependent macrophage inflammatory responses

**Fig. 4:**
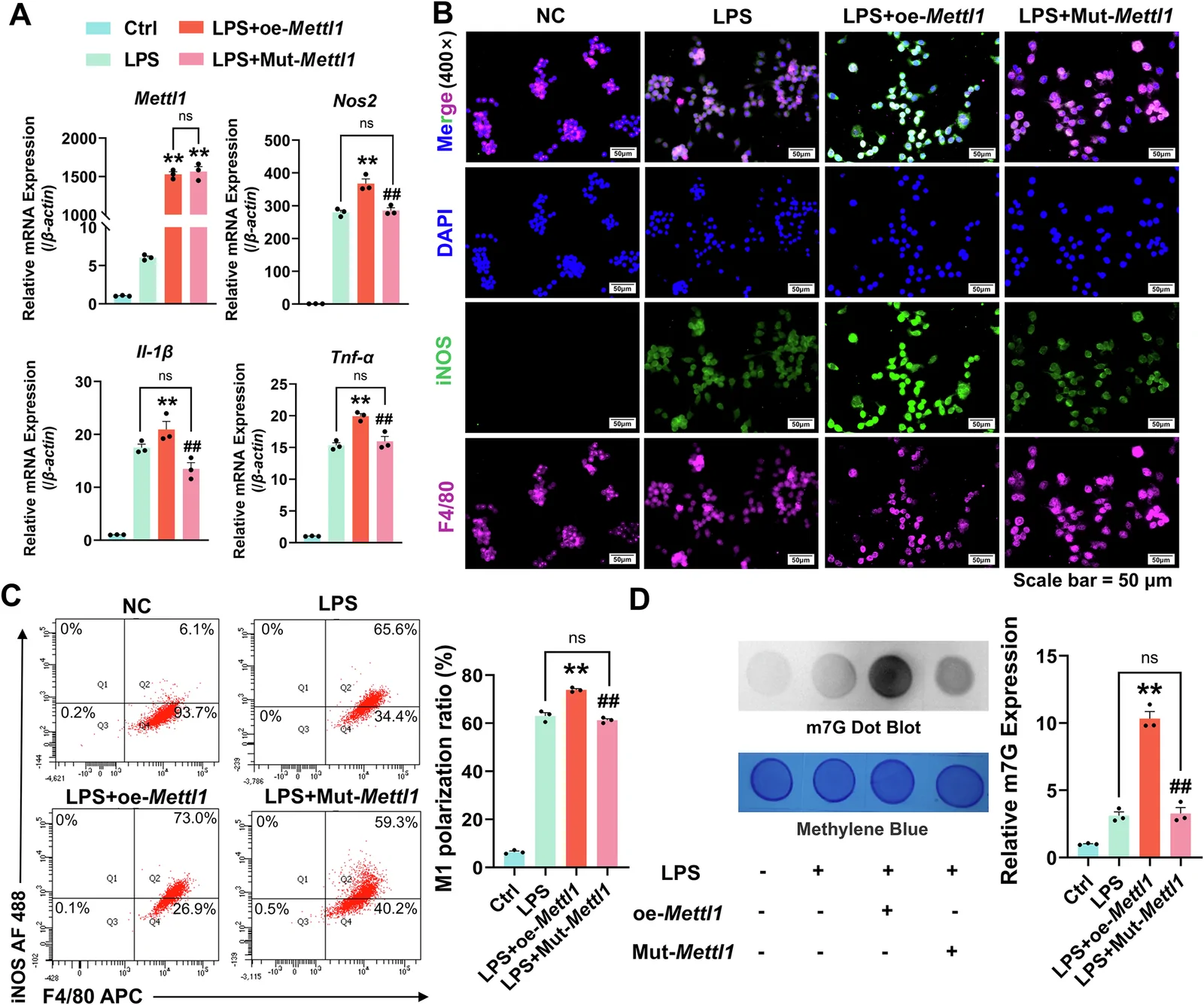
METTL1 catalytic activity is required for m7G-dependent macrophage inflammatory responses. **A** In LPS-stimulated macrophages, expression of catalytic-dead Mut-*Mettl1* attenuates M1-related gene expression, as determined by qPCR for *Mettl1, Nos2, Il-1β* and *Tnf-α*. **B** Immunofluorescence staining of F4/80 (magenta) and iNOS (green) with DAPI (blue) in NC, LPS, LPS+Mut-*Mettl1* and LPS+oe-*Mettl1* groups (400×). **C** Flow cytometry analysis and quantification of M1 polarization ratio (F4/80⁺iNOS⁺) in NC, LPS, LPS+Mut-*Mettl1* and LPS+oe-*Mettl1* (*n* = 3). **D** m7G dot blot of total RNA from macrophages under the indicated conditions (Ctrl, LPS+oe-*Mettl1*, LPS+Mut-*Mettl1*, LPS). Methylene blue staining serves as a loading control. Quantification of relative m7G levels from dot blot signals. Data are presented as mean ± SEM. **P < 0.01 vs. LPS, ^##^*P* < 0.01 vs. LPS+ oe-*Mettl1*.

To dissect whether METTL1 enzymatic activity is required for macrophage activation, we generated a catalytic-dead METTL1 mutant by substituting the conserved LFPD motif (aa160–163) with AFPA (Supplementary Fig. S1A, B) and confirmed successful expression of the Mut-GFP construct in macrophages (Supplementary Fig. S1C). In LPS-stimulated macrophages, the expression of Mut-*Mettl1* (validated by increased *Mettl1* transcripts) significantly attenuated the induction of M1 markers *Nos2, Il-1β*, and *Tnf-α* compared with LPS + oe-*Mettl1* (Fig. 4A). Consistently, Mut-*Mettl1* expression obviously downregulated the proportion of F4/80⁺ iNOS⁺ macrophages compared with LPS+oe-*Mettl1* treatment by immunofluorescence and flow cytometry (Fig. 4B, C). m7G dot blot demonstrated that overexpression of wild-type METTL1 enhanced global m7G levels in LPS-treated macrophages, whereas expression of the catalytic-dead mutant failed to increase m7G levels (Fig. 4D). These findings support that METTL1 promotes macrophage inflammatory activation in an m7G methyltransferase activity–dependent manner, providing mechanistic evidence linking METTL1-mediated m7G modification to AP-associated inflammation.

### *METTL1* overexpression reshapes the m7G methylation landscape

**Fig. 5:**
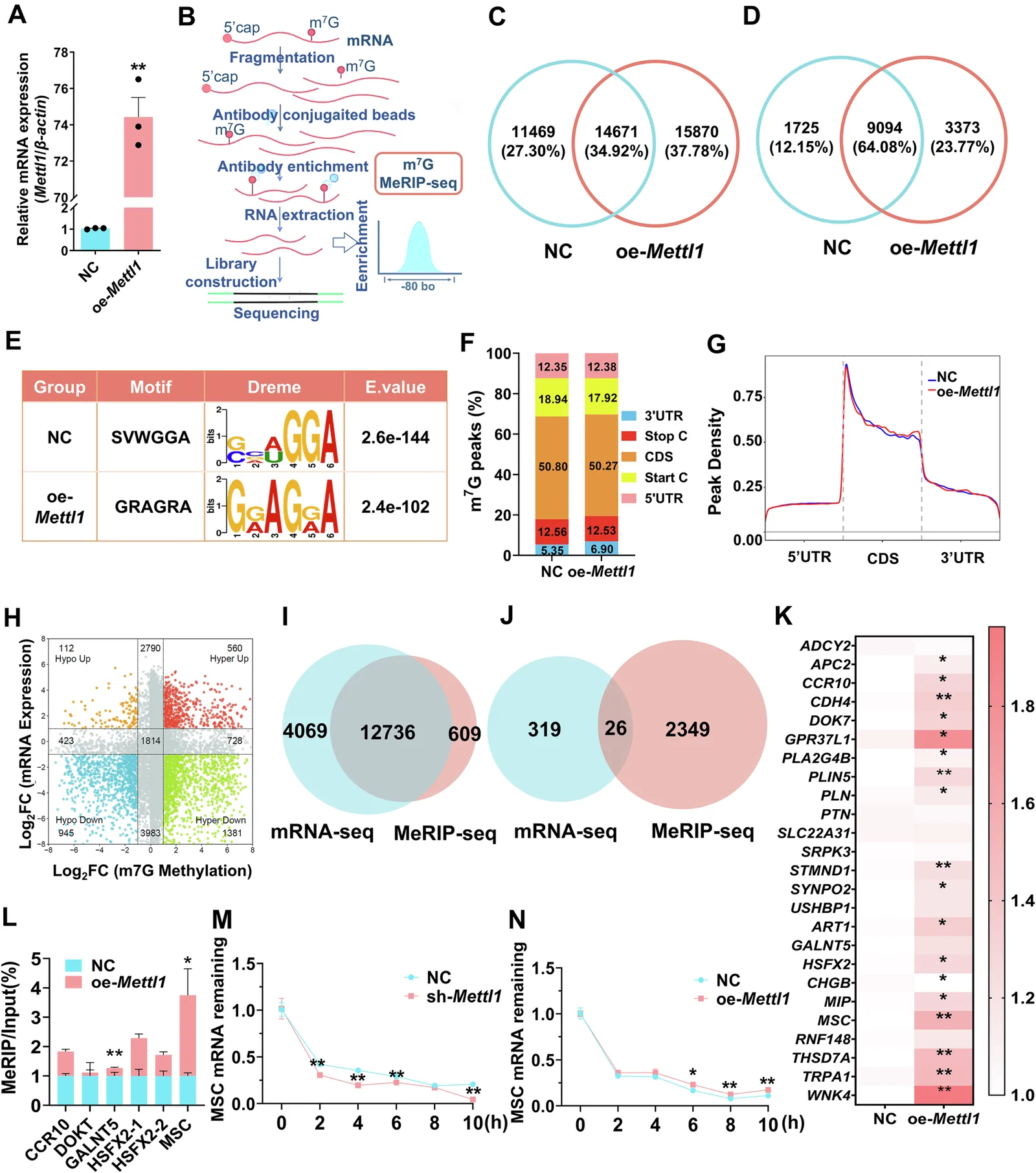
METTL1-mediated m7G methylation stabilizes MSC mRNA. **A** qPCR validation of *Mettl1* overexpression in 293 T cells. **B** Schematic overview of the m7G-MeRIP-seq workflow. **C** Venn diagram showing overlap and group-specific m7G peaks between NC and oe-*Mettl1* cells. **D** Venn diagram showing overlap and group-specific m7G-modified mRNAs between NC and oe-*Mettl1* cells. **E** DREME motif analysis of m7G peaks in NC and oe-*Mettl1* groups. **F** Distribution of m7G peaks across transcript regions (5′UTR, start codon region, CDS, stop codon region, and 3′UTR). **G** Metagene profiles showing m7G peak density along normalized transcript length (5′UTR, CDS, 3′UTR) in NC and oe-*Mettl1* groups. **H** Nine-quadrant plot showing the relationship between changes in m7G methylation (x-axis) and mRNA expression (y-axis). **I** Venn diagram showing the overlap of genes detected by mRNA-seq and MeRIP-seq. **J** Venn diagram showing the overlap between differentially expressed mRNAs (mRNA-seq) and differentially m7G-modified transcripts (MeRIP-seq), yielding 26 shared candidates. **K** qPCR validation of the 26 candidate genes identified by integrated analysis. **L** MeRIP qPCR analysis (MeRIP/Input, %) for representative targets (*CCR10, DOK7, GALNT5, HSFX2-1/2* and *MSC*) in NC and oe-*Mettl1* groups. **M**, **N** Actinomycin D chase assays showing MSC mRNA decay kinetics in sh-*Mettl1* vs. NC cells (**M**), and in oe-*Mettl1* vs. NC cells (**N**). Data are presented as mean ± SEM; *P < 0.05, **P < 0.01 vs. NC.

To delineate how METTL1 affects the m7G epitranscriptome, we established *Mettl1*-overexpressing 293 T cells (oe-*Mettl1*), as validated by qPCR (Fig. 5A), and performed m7G-MeRIP-seq (workflow shown in Fig. 5B). Global profiling revealed extensive remodeling of m7G peaks upon METTL1 overexpression: 14,671 peaks were shared between groups, while 15,870 peaks were unique to oe-*Mettl1* cells and 11,469 peaks were unique to NC cells (Fig. 5C). At the transcript level, 9,094 m7G-modified mRNAs were common to both groups, with 3,373 m7G-modified mRNAs specific to oe-*Mettl1* and 1,725 specific to NC (Fig. 5D). Motif discovery using DREME identified distinct enriched motifs between groups, with SVWGGA (S = C/G, V = A/C/G, W = A/U) enriched in NC and GRAGRA (R = G/A) enriched in oe-*Mettl1* cells (Fig. 5E), supporting a METTL1-dependent shift in m7G peak sequence context. We next mapped the distribution of m7G peaks along transcripts. m7G peaks were predominantly enriched within coding regions (CDS, ~50% in both groups), with modest redistribution across other compartments, including increased representation in the 3′UTR in oe-*Mettl1* cells (Fig. 5F). Metagene analysis further confirmed that m7G peaks were concentrated around translational regulatory regions across transcripts, with overall distribution patterns comparable between groups but altered peak density upon METTL1 overexpression (Fig. 5G).

### METTL1-mediated m7G modification stabilizes MSC mRNA

To link methylation changes with transcriptional output, we integrated MeRIP-seq with mRNA-seq and visualized the relationship between differential m7G methylation and differential gene expression using a nine-quadrant plot (Fig. 5H). This analysis highlighted subsets of genes showing concordant changes, including Hyper-Up and Hypo-Down clusters. Notably, Venn diagram showed the overlap of genes detected by mRNA-seq and MeRIP-seq (Fig. 5I), intersecting differentially expressed mRNAs with differentially m7G-modified transcripts yielded 26 shared candidates (Fig. 5J), which were further validated by qPCR, showing expression patterns consistent with sequencing results (Fig. 5K).

To identify direct METTL1/m7G-regulated targets, MeRIP qPCR was performed for representative candidates. Among the examined transcripts (*CCR10*, *DOK7*, *GALNT5*, *HSFX2-1/*2, and *MSC*), METTL1 overexpression produced the most prominent enrichment and upregulation of MSC (Fig. 5L). Given the role of mRNA modifications in transcript stability, we assessed MSC mRNA decay following actinomycin D treatment. MSC mRNA levels at each time point were quantified by qPCR, normalized to the internal control, and expressed relative to the 0 h time point. *Mettl1* knockdown accelerated MSC mRNA decay (reduced MSC mRNA remaining over time; Fig. 5M), whereas *Mettl1* overexpression delayed MSC decay and increased transcript longevity (Fig. 5N), indicating that METTL1 promotes MSC mRNA stability.

### MSC promotes macrophage M1 polarization via TNF signaling pathway

**Fig. 6:**
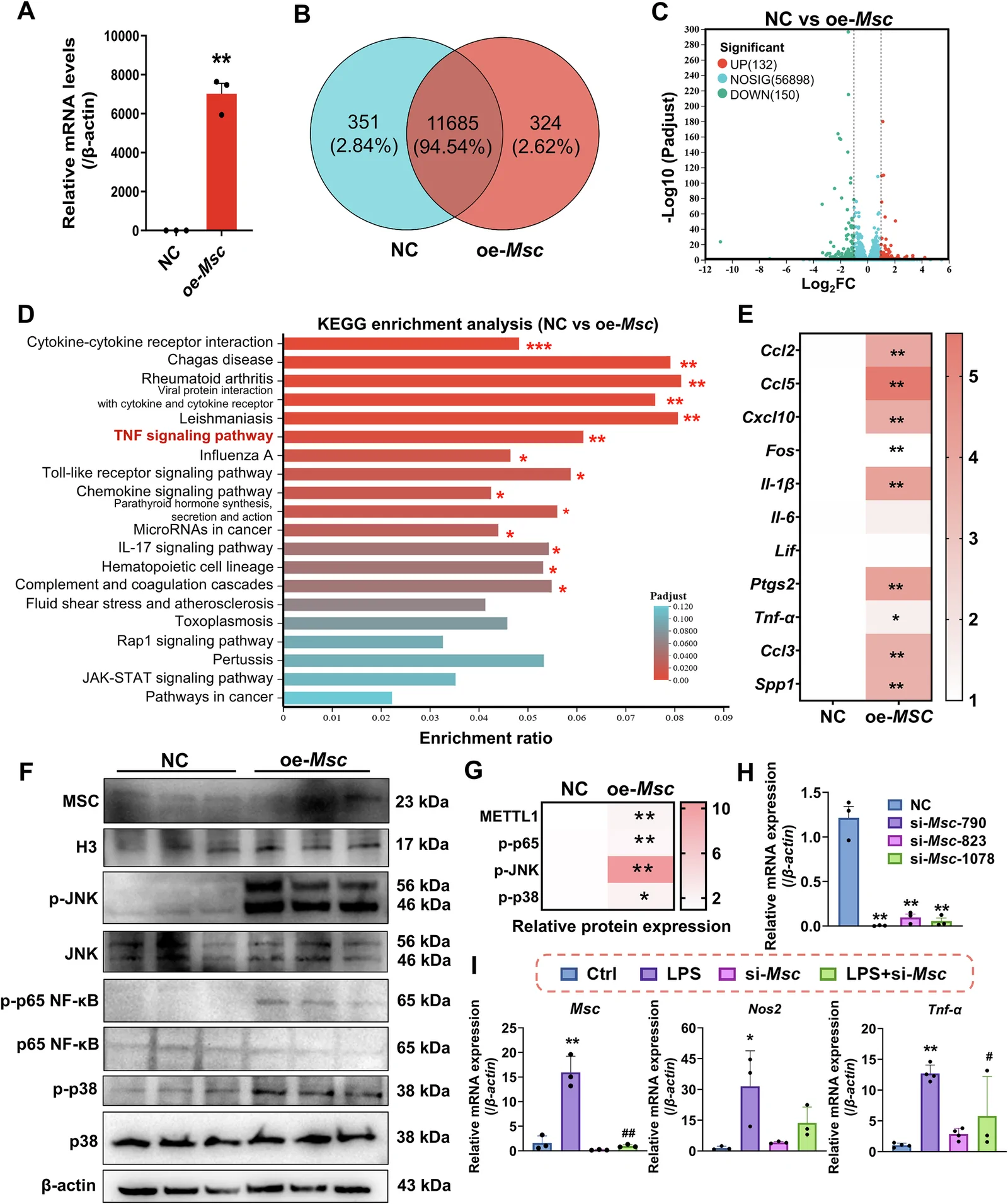
MSC promotes macrophage M1 polarization via TNF signaling pathway. **A** qPCR verification of *Msc* overexpression in macrophages. **B** Venn diagram showing overlap of detected genes between NC and oe-*Msc* macrophages. **C** Volcano plot of differentially expressed genes (DEGs) in oe-*Msc* versus NC macrophages (UP, 132; DOWN, 150; Padjust as indicated). **D** KEGG pathway enrichment analysis of DEGs between NC and oe-*Msc* groups; the TNF signaling pathway is highlighted. **E** qPCR validation of representative inflammation-related genes in NC and oe-*Msc* macrophages, including *Msc*, *Ccl2*, *Ccl5*, *Cxcl10*, *Fos*, *Il-1β*, *Il-6*, *Lif*, *Ptgs2*, *Tnf-α*, *Ccl3*, and *Spp1*. **F** Western blot analysis of MSC and TNF downstream signaling proteins in NC and oe-*Msc* macrophages, including phosphorylation of NF-κB p65, JNK, and p38 (p-p65/p65, p-JNK/JNK, p-p38/p38). **G** Relative quantification of the protein expression. **H** qPCR verification of Msc knockdown in macrophages. **I** qPCR validation of representative inflammation-related genes in macrophages (NC, LPS, si-*Msc* and LPS+si-*Msc)*, including *Msc*, *Nos2*, and *Tnf-α*. Data are presented as mean ± SEM; *P < 0.05, **P < 0.01 vs. NC. ^#^*P* < 0.05, ^##^*P* < 0.01 vs. LPS.

To investigate the mechanism by which MSC drives macrophage activation, we performed RNA-Seq in oe-*Msc* macrophages (Fig. 6A). The transcriptome profiles were highly overlapping between groups, with 11,685 commonly detected genes, while 351 and 324 genes were uniquely detected in NC and oe-*Msc*, respectively (Fig. 6B). Differential expression analysis identified 282 significantly altered genes in oe-*Msc* macrophages, including 132 upregulated and 150 downregulated genes (Fig. 6C). KEGG enrichment analysis of the differentially expressed genes revealed strong enrichment of inflammation-related pathways, prominently including the TNF signaling pathway, along with other immune signaling pathways such as Toll-like receptor and IL-17 signaling (Fig. 6D). Subsequently, qPCR confirmed that oe-*Msc* macrophages exhibited increased expression of multiple inflammatory mediators and response genes, including *Ccl2, Ccl5, Cxcl10, Fos, Il-1β, Il-6, Lif, Ptgs2, Tnf-α, Ccl3*, and *Spp1* (Fig. 6E). Consistent with the pathway analysis, western blotting showed robust MSC overexpression and increased activation of TNF downstream signaling, evidenced by elevated phosphorylation of NF-κB p65, JNK, and p38 in oe-*Msc* macrophages (Fig. 6F, G). Together, these data indicate that MSC promotes a pro-inflammatory transcriptional program in macrophages at least in part through activation of TNF-associated NF-κB/JNK/MAPK signaling. In addition, to explore the activity of MSC in macrophage polarization, we constructed siRNAs and validated the silenced efficiency using qPCR (Fig. 6H). Functionally, MSC depletion attenuated the LPS-induced upregulation of inflammatory/M1-associated genes, including *Nos2* and *Tnf-α*, in macrophages (Fig. 6I). These data demonstrate that METTL1 overexpression broadly reshapes the m7G methylation landscape and promotes inflammatory gene expression, at least in part through m7G-associated stabilization of MSC mRNA.

### TNF pathway inhibition attenuates METTL1/MSC-driven macrophage activation

**Fig. 7:**
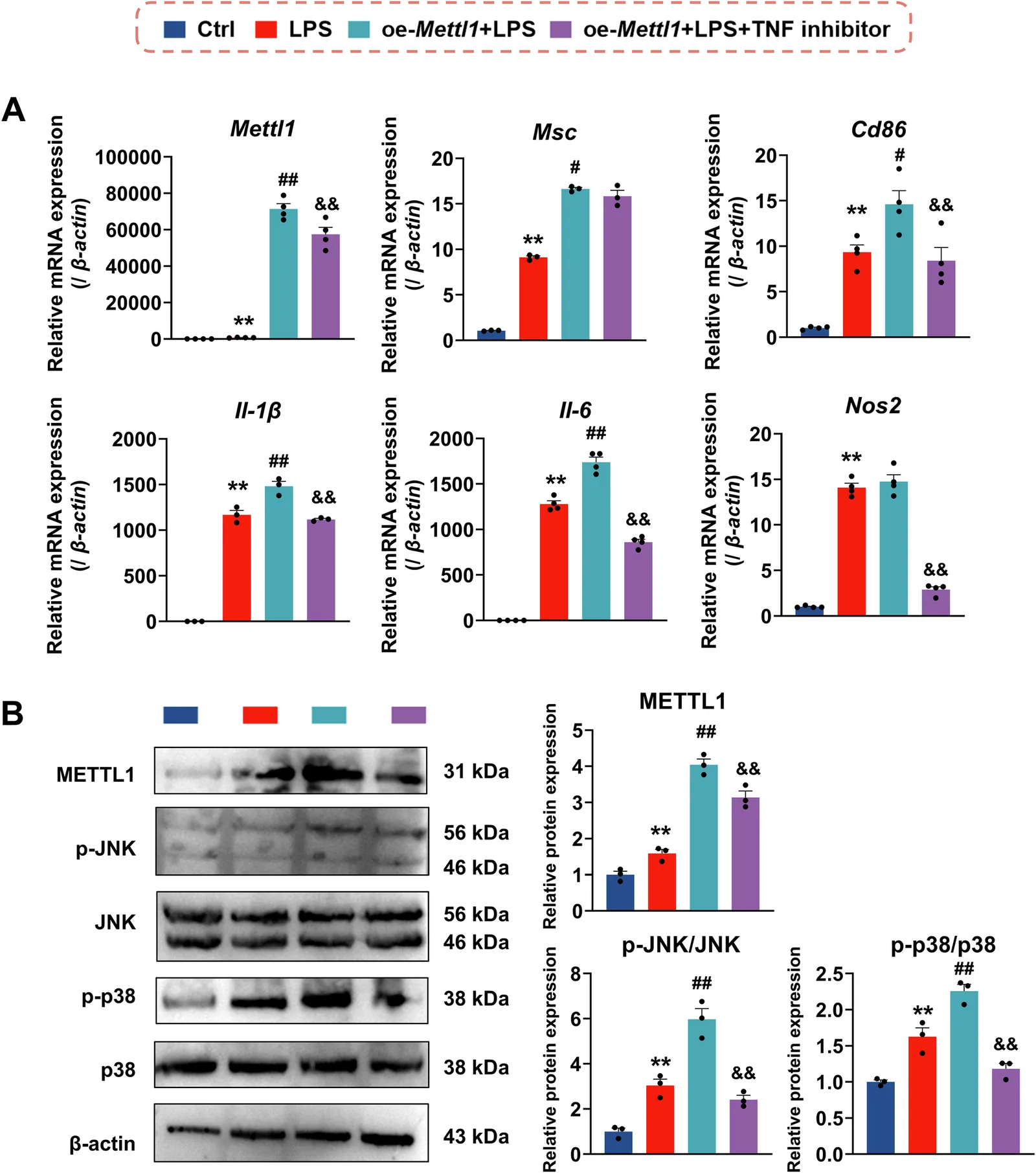
TNF pathway inhibition attenuates METTL1/MSC-driven macrophage activation. **A** qPCR analysis of *Mettl, Msc, Cd86, Il-1β, Il-6* and *Nos2* expression in macrophages (Ctrl, LPS, LPS+oe-*Mettl1* and LPS+oe-*Mettl1* + TNF inhibitor). **B** Western blot analysis of METTL1, JNK, and p38 (p-JNK/JNK, p-p38/p38) protein in macrophages with β-actin as loading control and densitometric quantification. Data are presented as mean ± SEM; **P < 0.01 vs. Ctrl, ^#^*P* < 0.05, ^##^*P* < 0.01 vs. LPS ^& &^*P* < 0.01 vs. LPS+oe-*Mettl1*.

To validate the functional involvement of TNF signaling in METTL1-mediated macrophage activation, RAW264.7 cells were stimulated with LPS, with or without *Mettl1* overexpression, and an additional group (oe-*Mettl1* + LPS) was treated with a TNF pathway inhibitor. LPS stimulation increased the expression of *Mettl1* and M1-associated markers, including *Il-1β, Il-6, Nos2* and *Cd86*, and these responses were further enhanced in oe-*Mettl1* + LPS cells, accompanied by a marked induction of *Msc*. Notably, pharmacologic blockade of TNF signaling in the oe-*Mettl1* + LPS context significantly dampened the upregulation of *Il-1β, Il-6, Nos2*, and *Cd86*, whereas *Mettl1* expression remained elevated (Fig. 7A). Consistently, western blot showed that LPS—and more prominently oe-*Mettl1* + LPS—enhanced phosphorylation of JNK and p38 MAPK, while TNF inhibitor markedly decreased p-JNK/JNK and p-p38/p38 ratios without appreciably altering total JNK or p38 levels (Fig. 7B). Together, these data support that activation of the TNF/JNK/p38 axis is required for METTL1/MSC-driven pro-inflammatory activation in macrophages.

### METTL1/m7G methylation exacerbates PACs injury through MSC-TNF pathway

**Fig. 8:**
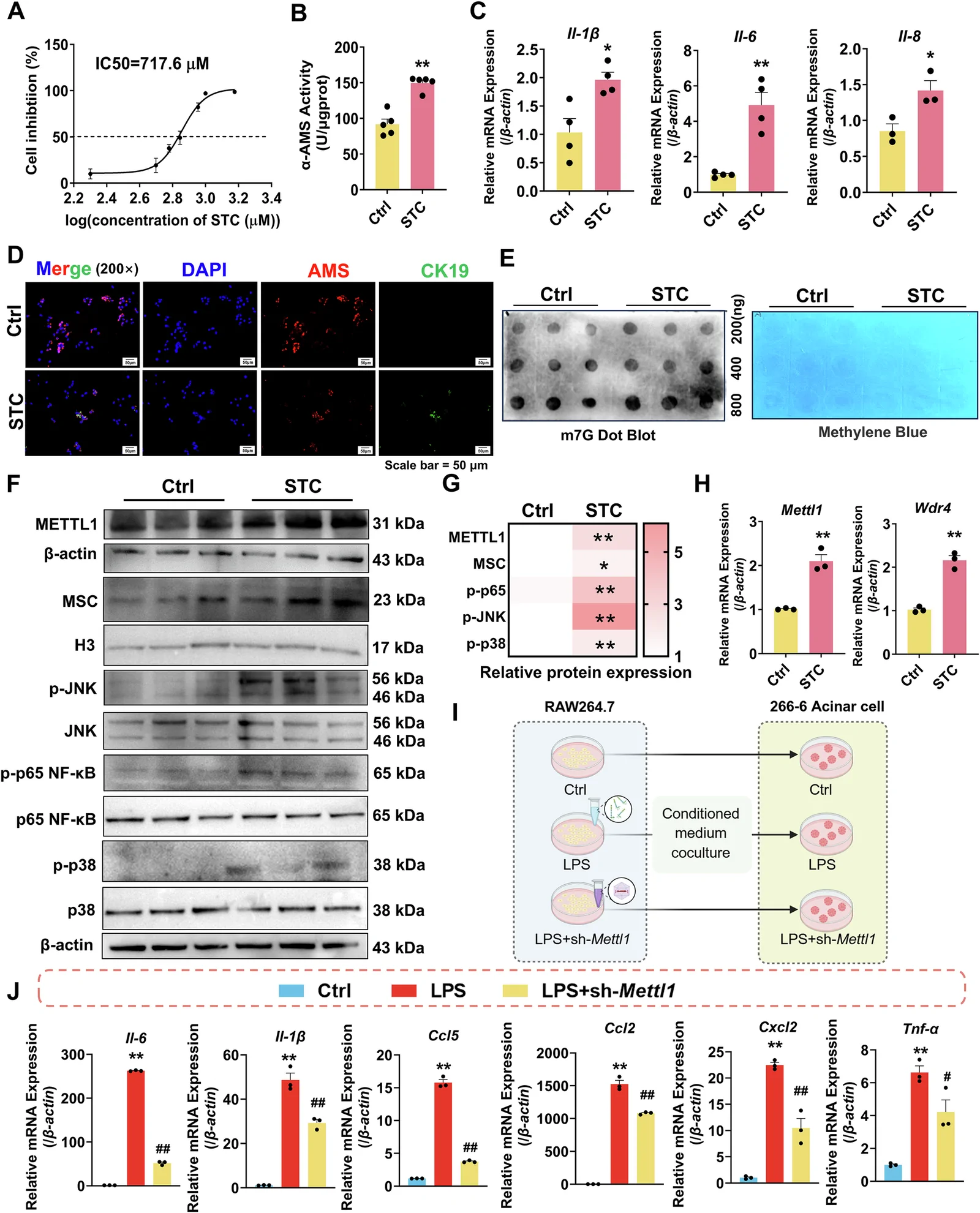
METTL1/m7G methylation exacerbates PACs injury through MSC-TNF pathway. **A** CCK-8 assay showing STC dose–response and calculated IC_50_ in 266-6 acinar cells. **B** The α-amylase activity in culture supernatants of 266-6 cells treated with STC or vehicle control. **C** qPCR analysis of *Il-1β, Il-6* and *Il-8* mRNA levels in Ctrl and STC-treated 266-6 cells. **D** Immunofluorescence images of 266-6 acinar cells following STC stimulation. Cells were stained for amylase (red), CK19 (green), and nuclei were counterstained with DAPI (blue). **E** m7G dot blot analysis of global m7G levels in total RNA from Ctrl and STC-treated 266-6 cells, with methylene blue staining as loading control. **F** Western blot analysis of METTL1, MSC and TNF downstream signaling proteins (p-p65/p65, p-JNK/JNK, p-p38/p38) with β-actin as loading control and densitometric quantification in Ctrl and STC-treated 266-6 cells. **G** Quantification of phosphorylation ratios (METTL1/β-actin, MSC/H3, p-p65/p65, p-JNK/JNK, p-p38/p38). **H** qPCR analysis of *Mettl1* and *Wdr4* mRNA levels in Ctrl and STC-treated 266-6 cells. **I** Schematic of conditioned-medium co-culture, RAW264.7 macrophages (Ctrl, LPS, LPS+sh-*Mettl1*) conditioned media applied to 266-6 acinar cells. **J** qPCR analysis of inflammatory genes in 266-6 cells after conditioned-medium treatment, *Il-1β, Il-6, Tnf-α, Ccl2, Ccl5* and *Cxcl2*. Data are presented as mean ± SEM. *P < 0.05, **P < 0.01 vs. Ctrl, ^*#*^*P* < 0.05, ^*##*^*P* < 0.01 vs. LPS.

Given that PACs injury represents a key initiating event in AP, we next examined whether METTL1-mediated m7G modification participates in STC-induced damage in 266-6 acinar cells. A CCK-8 dose–response assay showed that STC inhibited cell viability with an IC₅₀ of ~717.6 μM (Fig. 8A). Consistent with acinar injury, STC treatment significantly increased α-amylase activity in the culture supernatant (Fig. 8B) and elevated the mRNA levels of pro-inflammatory cytokines *Il-1β, Il-6*, and *Il-8* (Fig. 8C). Compared with the Ctrl group, STC-treated cells exhibited a marked reduction in amylase expression accompanied by increased CK19 signal intensity, indicating acinar cells undergo ductal metaplasia in response to external stimuli (Fig. 8D). Dot blot analysis further revealed a robust increase in global m7G levels following STC stimulation (Fig. 8E). In parallel, western blot confirmed increased METTL1 and its downstream effector MSC, accompanied by enhanced activation of TNF-associated signaling cascades, as indicated by increased phosphorylation of NF-κB p65, JNK, and p38 (Fig. 8F, G). qPCR showed that STC upregulated *Mettl1* and its cofactor Wdr4 in 266 cells (Fig. 8H). These data suggest that STC-induced PACs injury is coupled to increased METTL1/m7G signaling, MSC upregulation, and activation of inflammatory kinase pathways.

To determine whether macrophage METTL1 also modulates PACs inflammatory responses via soluble mediators, we established a conditioned-medium co-culture system (Fig. 8I). The 266-6 cells were incubated with supernatants from RAW264.7 macrophages under three conditions: Ctrl, LPS, and LPS with *Mettl1* knockdown (LPS+sh-*Mettl1*). Conditioned medium from LPS-activated macrophages markedly induced inflammatory gene expression in 266-6 cells, including *Il-1β*, *Il-6*, *Tnf-α*, *Ccl2*, *Ccl5*, and *Cxcl2* (Fig. 8J). Importantly, these pro-inflammatory effects were substantially attenuated when *Mettl1* was silenced in macrophages, indicating that macrophage METTL1-dependent factors potentiate inflammatory activation of PACs. Collectively, these findings support that METTL1-driven m7G signaling aggravates PACs injury both cell-autonomously (via MSC and downstream inflammatory signaling) and non–cell-autonomously through macrophage-derived paracrine inflammation.

## Discussion

Previous investigations mostly focused on the involvement of m7G in tumorigenesis, a recent study revealed its critical role in ulcerative colitis (UC)-associated intestinal inflammation, underscoring for the first time its broader implications in inflammatory diseases [16–18]. Here, we identify a previously underappreciated role of the METTL1/WDR4-associated m7G axis [19, 20] in AP. We observed elevated METTL1 expression and increased global m7G signals in STC-induced AP pancreatic tissues, in LPS-driven M1-like macrophages, and in STC-injured acinar cells. Importantly, we also detected significantly increased serum METTL1 levels in AP patients, supporting the clinical relevance of METTL1 dysregulation and suggesting its potential as a biomarker candidate for inflammatory pancreatic injury.

AP progression is tightly coupled to macrophage-driven inflammatory cascades and pancreatic tissue damage [20, 21]. In the early phase of AP, DAMPs and cytokine cues released by damaged-PACs reshape the pancreatic microenvironment [7], promoting macrophage recruitment and polarization toward a pro-inflammatory M1-like state [22–24]. Sustained activation of M1-like macrophages amplifies inflammatory mediator release and exacerbates tissue injury [25]. Consistent with this framework, LPS-induced M1 polarization together with increased METTL1 expression and elevated global m7G levels. METTL1 overexpression further enhanced the expression of M1-associated markers and inflammatory cytokines, but reversed by the silence of *Mettl1*, indicating that METTL1/m7G signaling is not merely a bystander but actively reinforces the pro-inflammatory macrophage phenotype. The in vivo significance of METTL1 in AP is further supported by our pancreas-targeted AAV-mediated knockdown model. Suppressing Mettl1 markedly alleviated AP severity, evidenced by improved histopathology, reduced serum digestive enzyme release, and attenuated inflammatory mediator induction. These findings provide functional evidence that METTL1 contributes to AP-associated pancreatic injury and inflammation in vivo. Together with the patient serum data, this in vivo validation strengthens the translational implication that METTL1 is positioned upstream of key inflammatory outputs during AP.

Mechanistically, we profiled the METTL1-regulated m7G epitranscriptome using m7G-MeRIP-seq coupled with RNA-seq. METTL1 overexpression reshaped the m7G landscape, generating extensive group-specific peaks and m7G-modified transcripts, and motif analysis revealed distinct enriched sequence contexts between control and oe-Mettl1 conditions. In addition, m7G peaks displayed a preferential distribution within coding and translation-related transcript regions, with measurable redistribution among transcript compartments. These global features suggest that METTL1 does not simply increase m7G abundance but can remodel m7G deposition patterns across the transcriptome, potentially influencing a coordinated set of inflammatory and stress-response programs.

By integrating differential m7G methylation with differential gene expression, we identified a subset of candidates exhibiting coordinated methylation and expression changes. Among these, MSC emerged as a prominent METTL1/m7G-regulated target, with MeRIP qPCR enrichment and actinomycin D chase assays supporting a METTL1-dependent increase in MSC mRNA stability. Musculin (MSC) is a conserved basic helix–loop–helix transcriptional regulator implicated in lymphocyte differentiation and immune programs [26, 27]. While MSC has been linked to B-cell fate decisions and inflammation-related phenotypes in other contexts, its role in macrophage-mediated innate immune responses has remained largely unexplored. Our findings place MSC downstream of METTL1/m7G signaling as a key node that contributes to macrophage inflammatory activation. To define downstream pathways engaged by MSC, RNA-seq of MSC-overexpressing macrophages revealed enrichment of inflammation-related signaling pathways, prominently including the TNF signaling pathway, and qPCR validation confirmed upregulation of representative TNF-associated genes. TNF signaling is central to innate immunity and has been implicated in AP severity through activation of NF-κB and MAPK cascades [28, 29]. TNF-α is known to correlate with pancreatic injury and can amplify inflammation by triggering p38/JNK activation and NF-κB-dependent transcription of pro-inflammatory mediators [30–32]. In line with this, MSC overexpression increased phosphorylation of NF-κB p65, JNK and p38 MAPK. Crucially, pharmacologic inhibition of TNF signaling attenuated METTL1/MSC-driven inflammatory gene induction and reduced downstream kinase phosphorylation, providing functional evidence that the TNF–JNK/p38 MAPK arm is required for METTL1/MSC-mediated macrophage activation. In addition to pathway inhibition, our catalytic-dead mutant experiments provide a stringent mechanistic test of enzymatic dependency. We generated a METTL1 mutant by disrupting the conserved catalytic motif and confirmed its expression in macrophages. Unlike wild-type METTL1, the catalytic-dead mutant failed to elevate global m7G signals and was insufficient to sustain robust induction of inflammatory/M1-associated genes under inflammatory stimulation. These results support that the pro-inflammatory effects of METTL1 depend on its m7G methyltransferase activity, strengthening the causal link between epitranscriptomic modification and macrophage inflammatory programming.

Beyond macrophages, pancreatic acinar cells (PACs) comprise the dominant cellular component of the pancreas and are highly susceptible to injury during AP [33]. In our STC-induced acinar injury model, we observed increased global m7G levels, upregulated METTL1/WDR4 and MSC expression, and activation of TNF-associated signaling, suggesting that METTL1/m7G signaling may participate in acinar inflammatory responses as well. Moreover, conditioned-medium experiments support a paracrine mechanism whereby macrophage METTL1 amplifies inflammatory activation of acinar cells, reinforcing reciprocal macrophage–acinar crosstalk. Collectively, these cell-autonomous and non–cell-autonomous observations provide a coherent explanation for how METTL1-driven m7G modification can propagate and amplify inflammatory injury during AP.

Several limitations warrant consideration. First, although our data support MSC stabilization as a key downstream event, pinpointing the precise functional m7G site(s) on MSC and defining the full spectrum of disease-relevant METTL1 targets in macrophages and acinar cells will require higher-resolution mapping and cell-type–specific approaches. Second, while AAV-mediated knockdown and pharmacologic TNF blockade support causality and pathway dependency, future studies employing cell-type–restricted genetic models will be valuable to disentangle macrophage- versus acinar-specific contributions. Despite these limitations, our findings support an integrated model in which METTL1-mediated m7G deposition stabilizes MSC mRNA, amplifies TNF-associated NF-κB/JNK/p38 signaling, promotes macrophage M1-like activation, and exacerbates acinar injury, forming a self-reinforcing inflammatory circuit in AP. This mechanistic framework, together with elevated serum METTL1 in AP patients, highlights METTL1 as a promising epitranscriptomic target for therapeutic intervention in AP.

## Conclusion

This study identifies a METTL1–m7G–MSC–TNF signaling axis as an inflammatory amplifier in acute pancreatitis. These findings provide epitranscriptomic evidence for m7G involvement in AP and nominate METTL1 as a promising target. While these findings elucidate novel mechanisms, further studies are required to fully delineate the role of m7G-modified MSC in AP-associated tissue damage and inflammation.

## Materials and methods

Additional experimental details, including AAV9-mediated *Mettl1* knockdown in mice, AP mouse model, enzymatic detection, hematoxylin and eosin staining, immunohistochemistry and immunofluorescence, Clinical sample collection, cell culture, cell viability, flow cytometry, dot blot, plasmid transfection, m7G-MeRIP sequencing, m7G-MeRIP qPCR, RNA sequencing, qPCR, western blot, cell co-culture and statistical analysis are provided in the Supplementary Information.

## Supplementary information

## Data Availability

All data in this study are provided in the paper and Supplementary Materials.
