# Supplementary material for: Methyltransferase METTL1 regulates MSC mRNA stability via m7G modification in acute pancreatitis
Source: Cell Death Dis. 2026 Jun 23;17(1):775. doi: 10.1038/s41419-026-09002-7 (PMC13538475; doi:10.1038/s41419-026-09002-7)
Supplement: Supplementary file 2 [file 41419_2026_9002_MOESM3_ESM.pdf]

Western blot

Figure 1

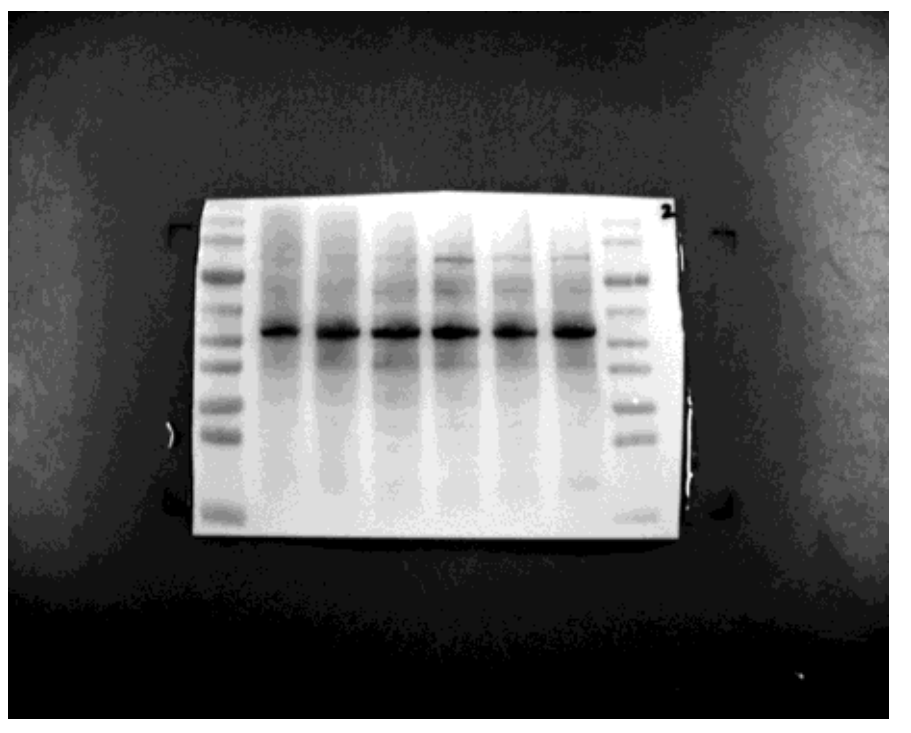

β-actin

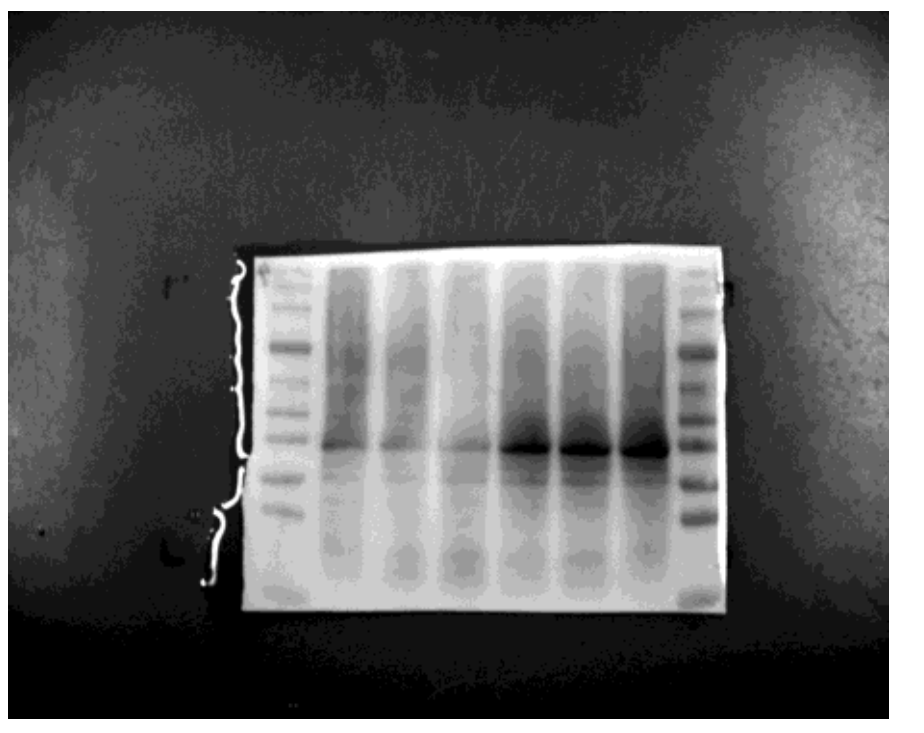

METTL1

Figure 2

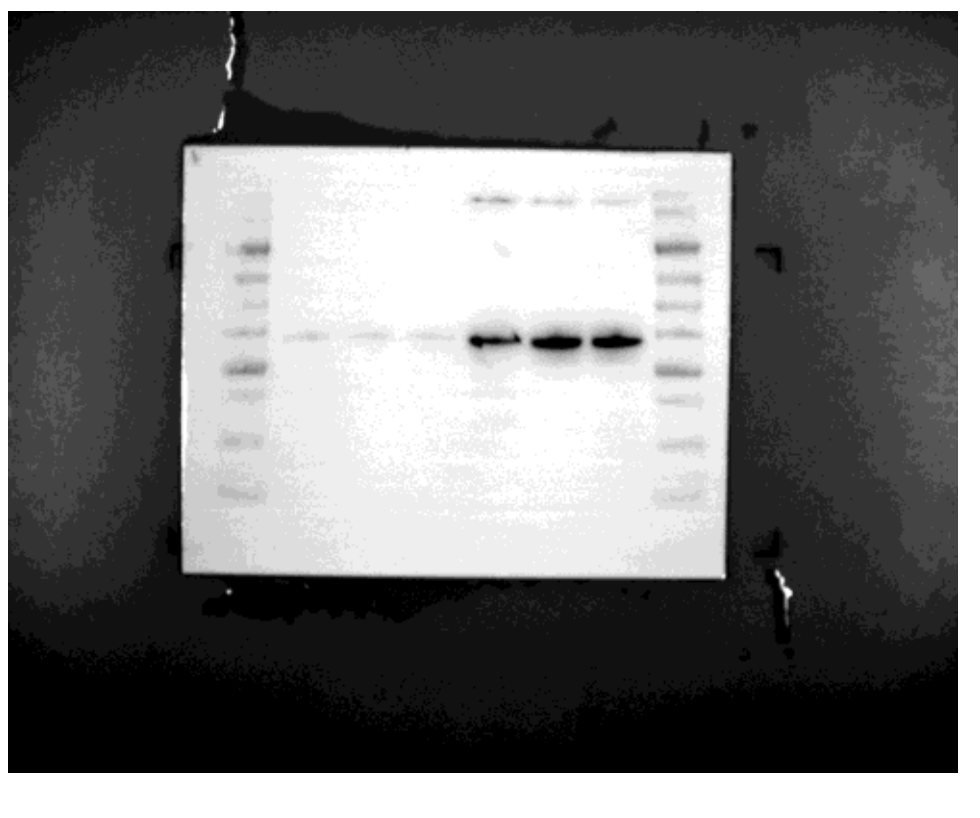

β-actin

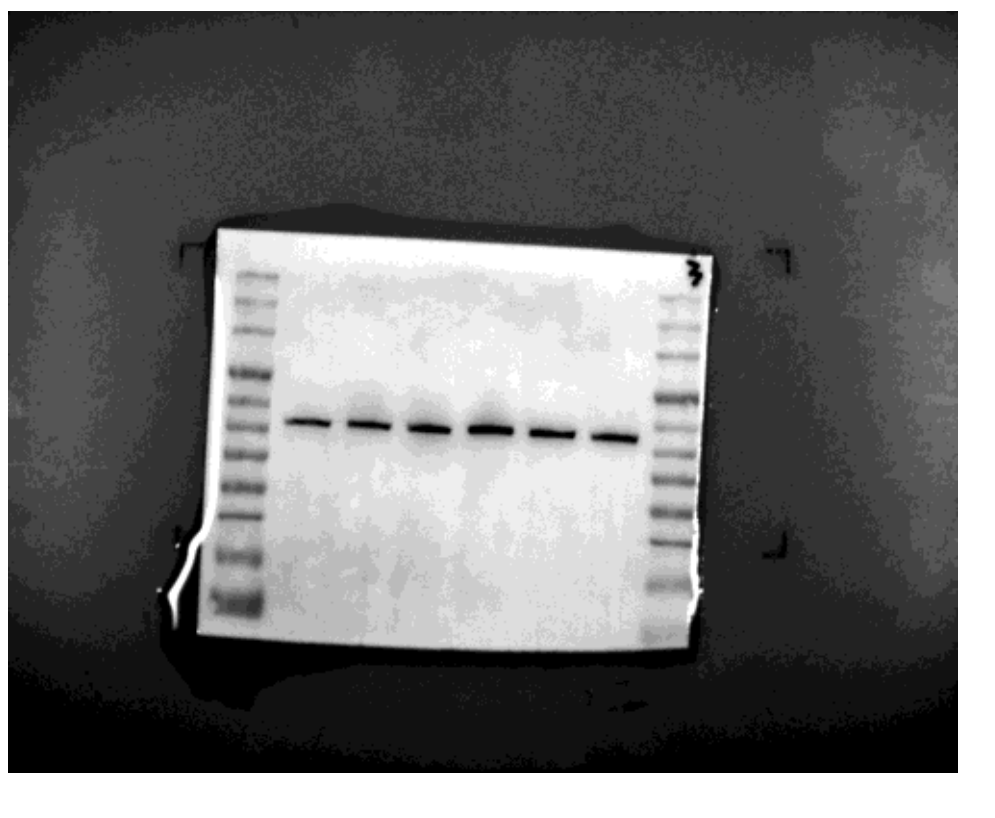

METTL1

Western blot

Figure 6

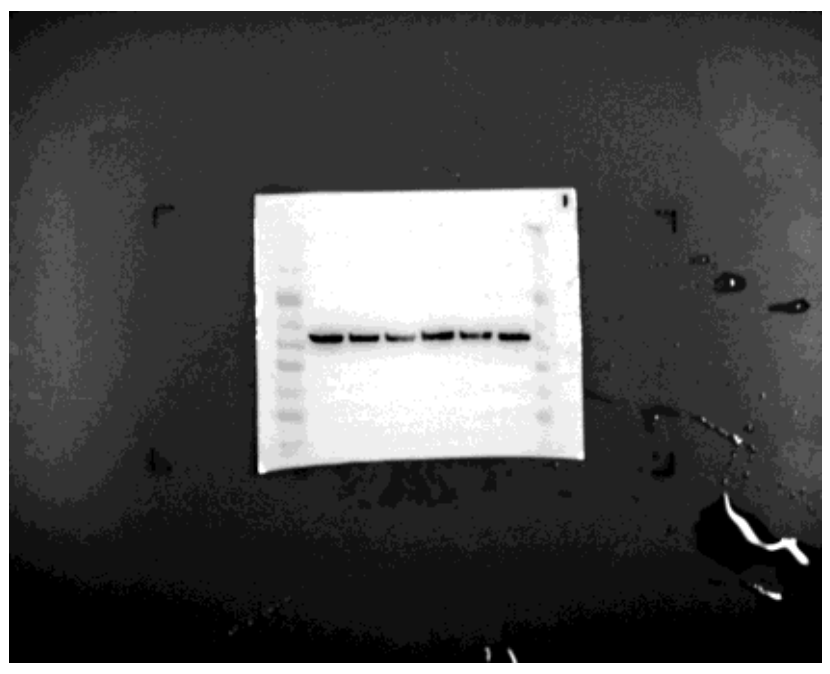

β-actin

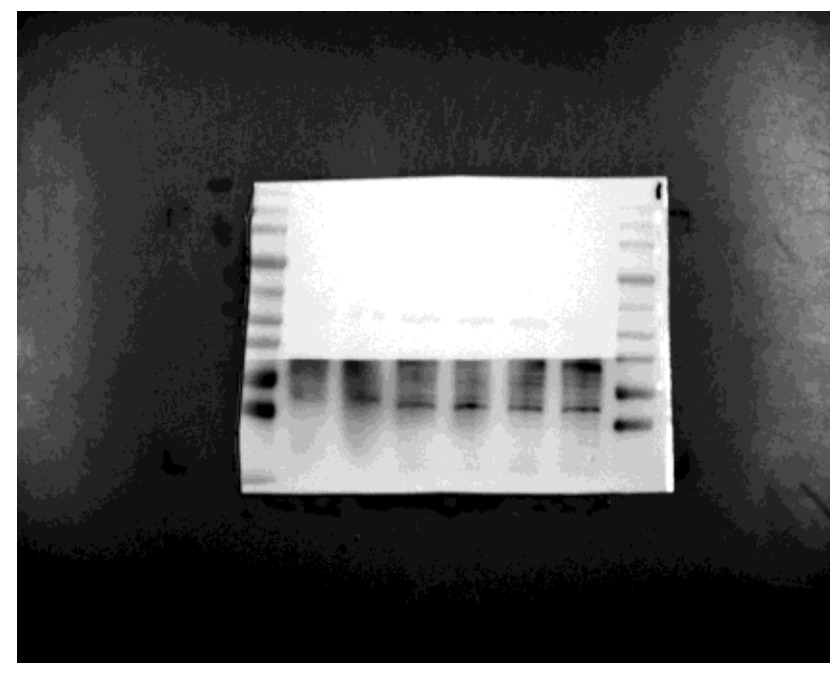

H3

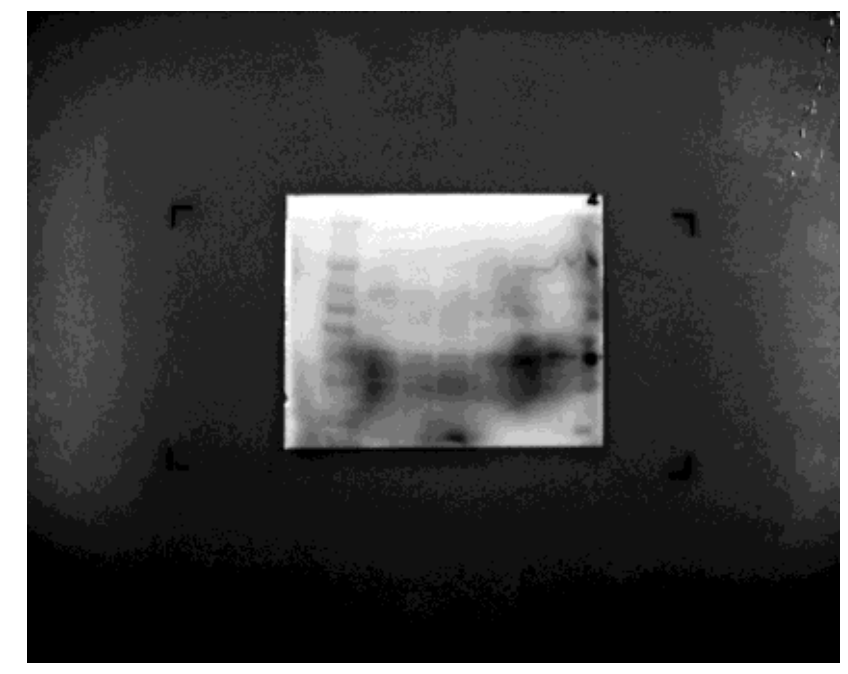

MSC

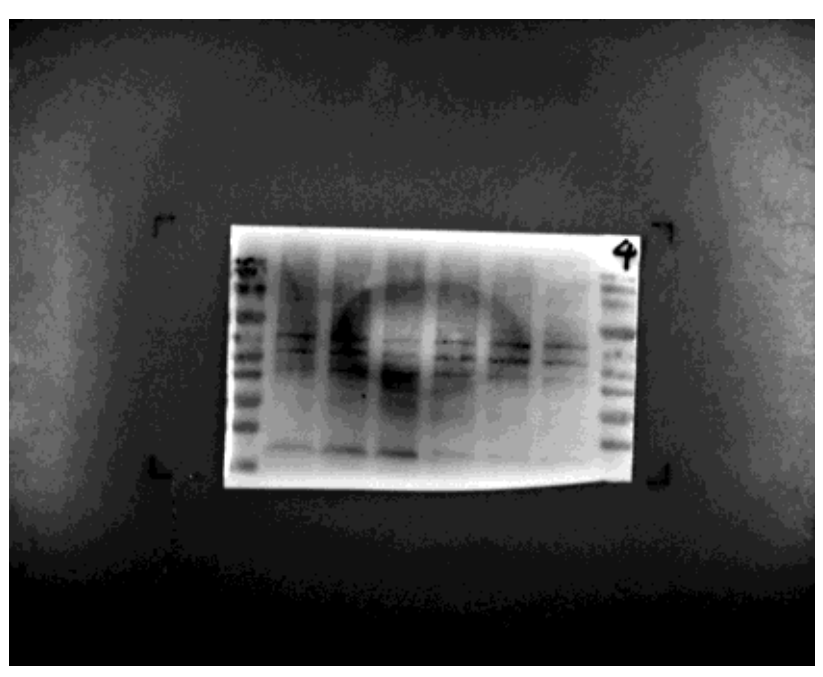

JNK

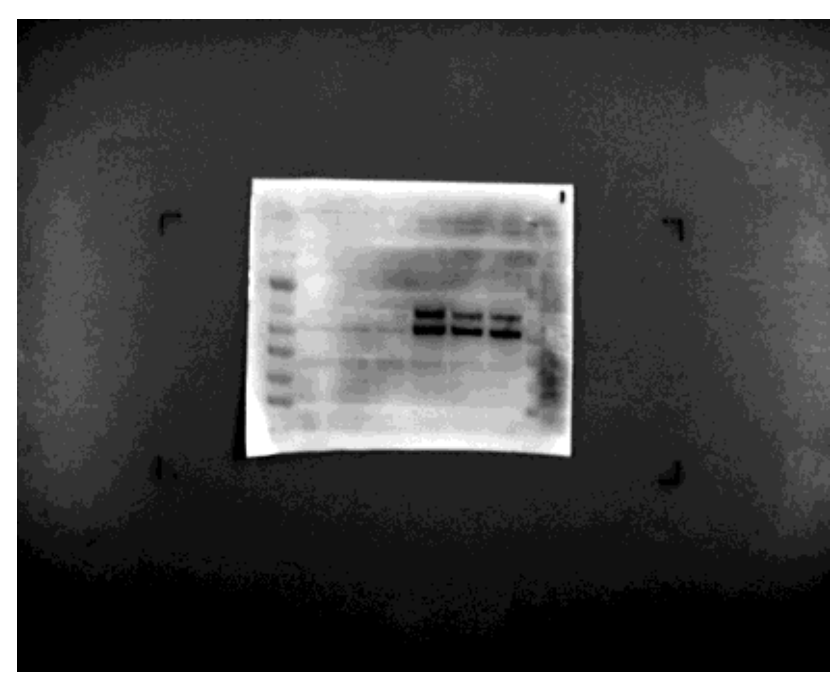

p-JNK

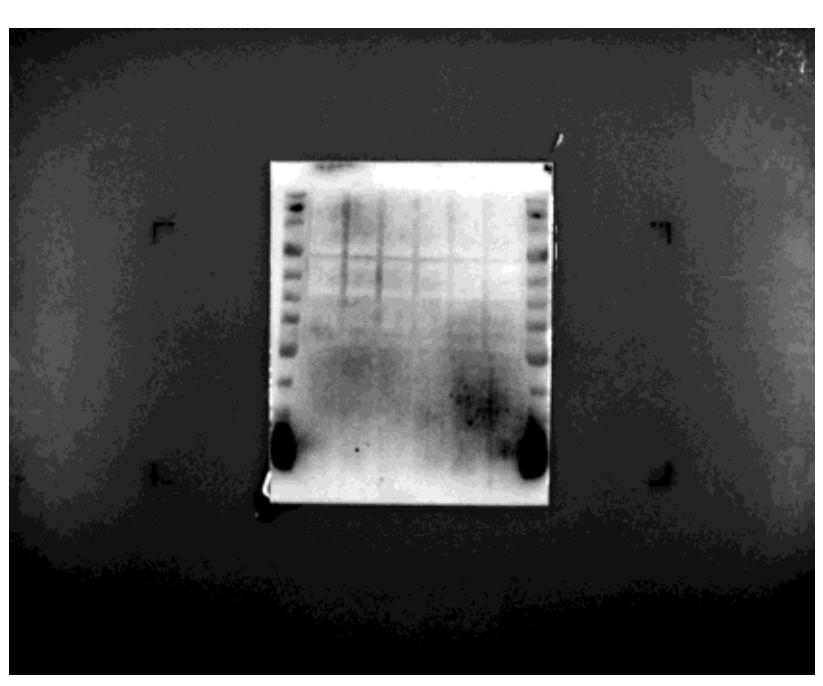

p65

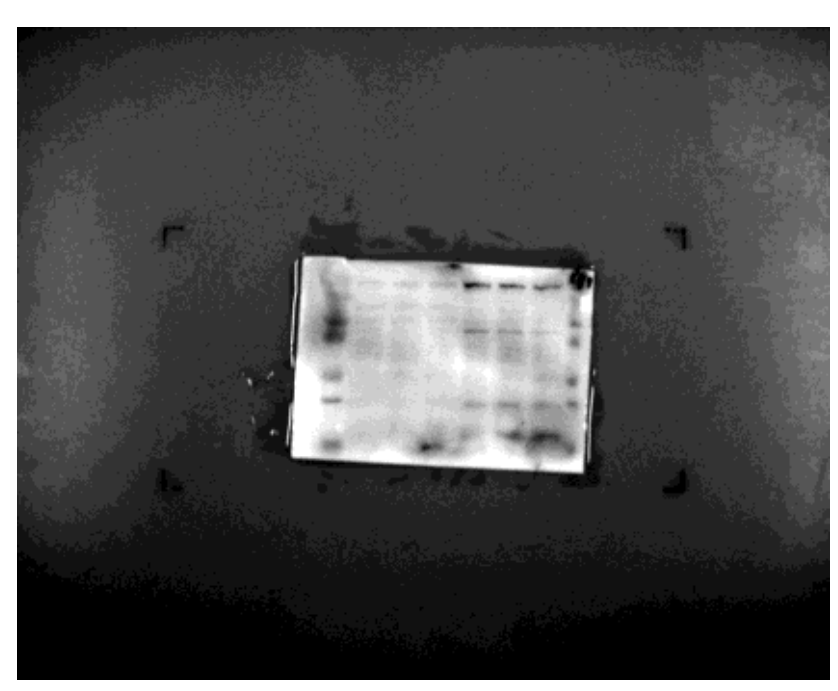

p-p65

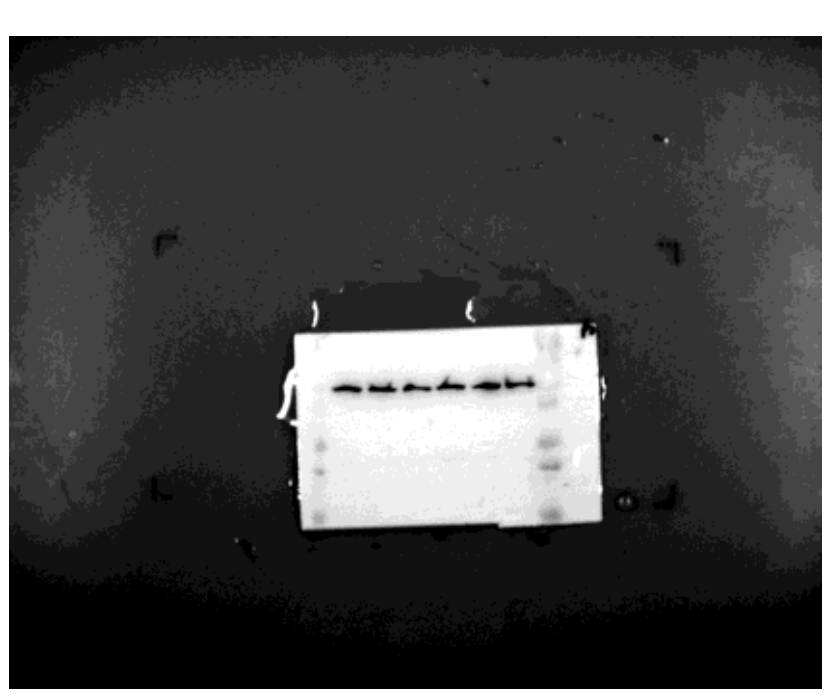

p38

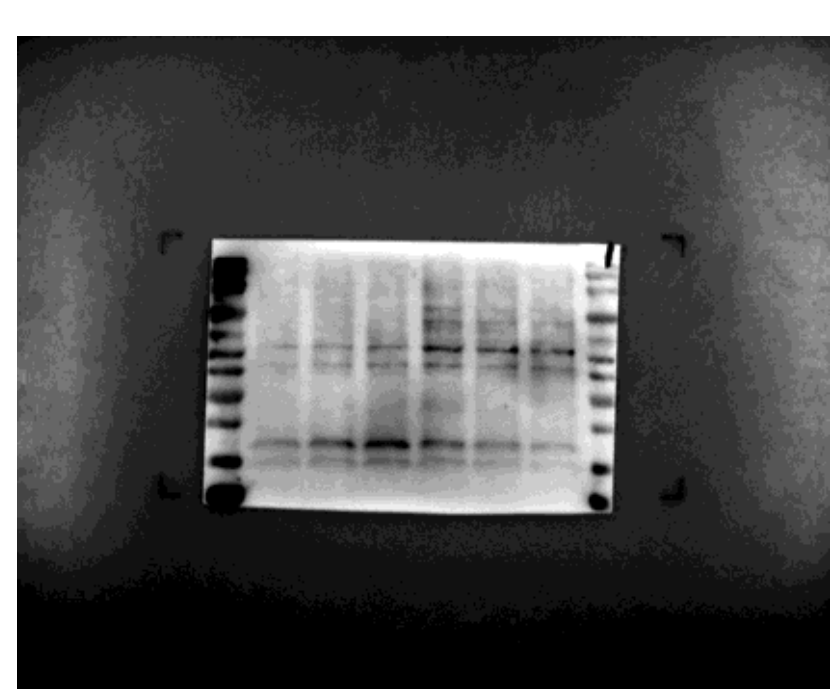

p-p38

Western blot

Figure 7

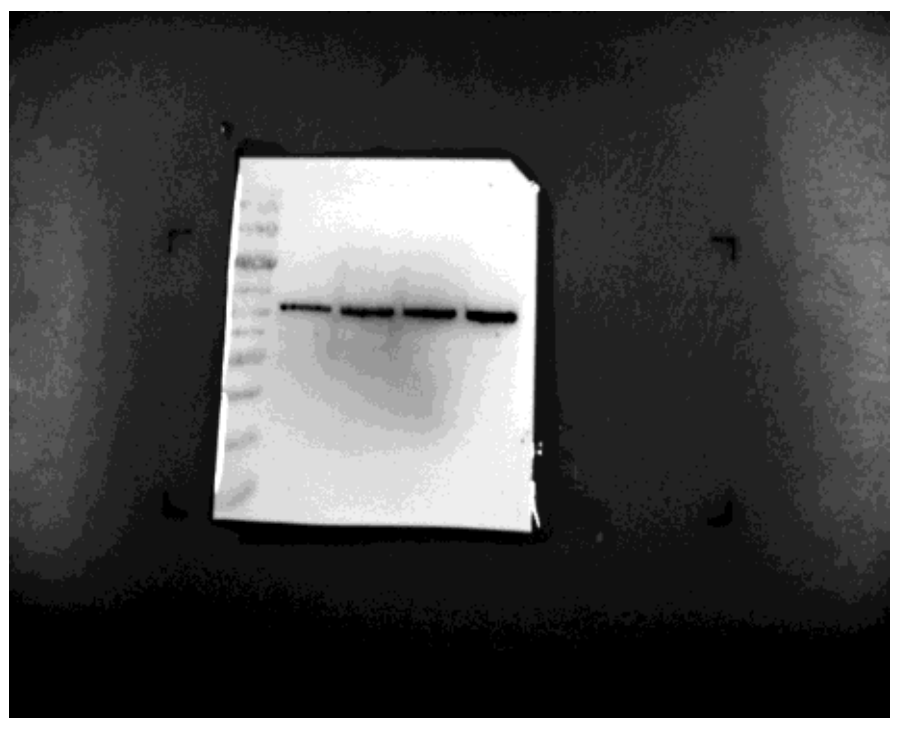

β-actin

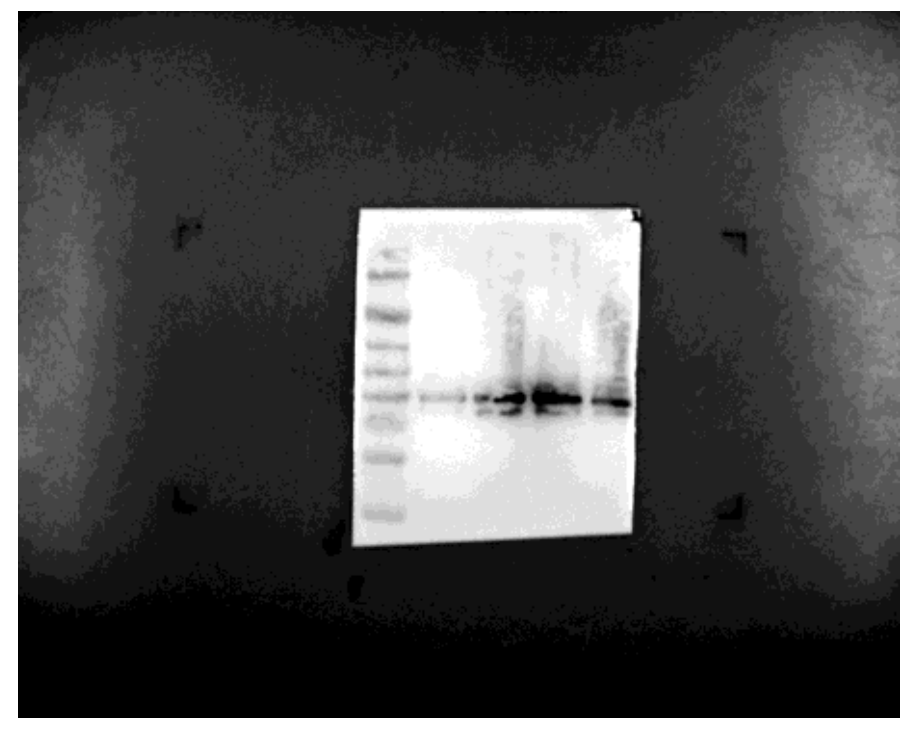

METTL1

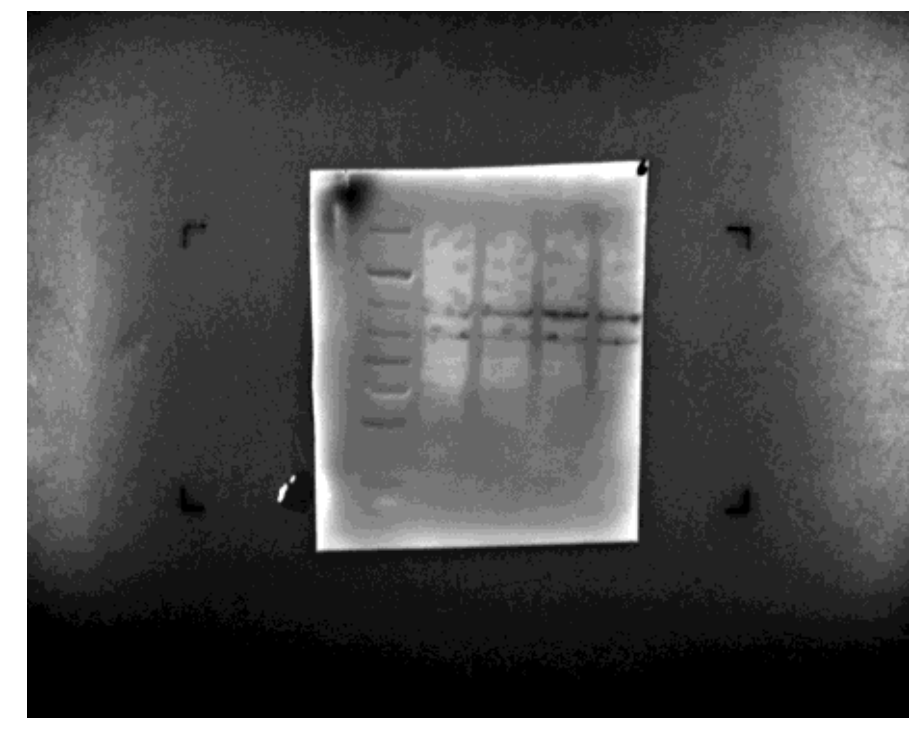

p-JNK

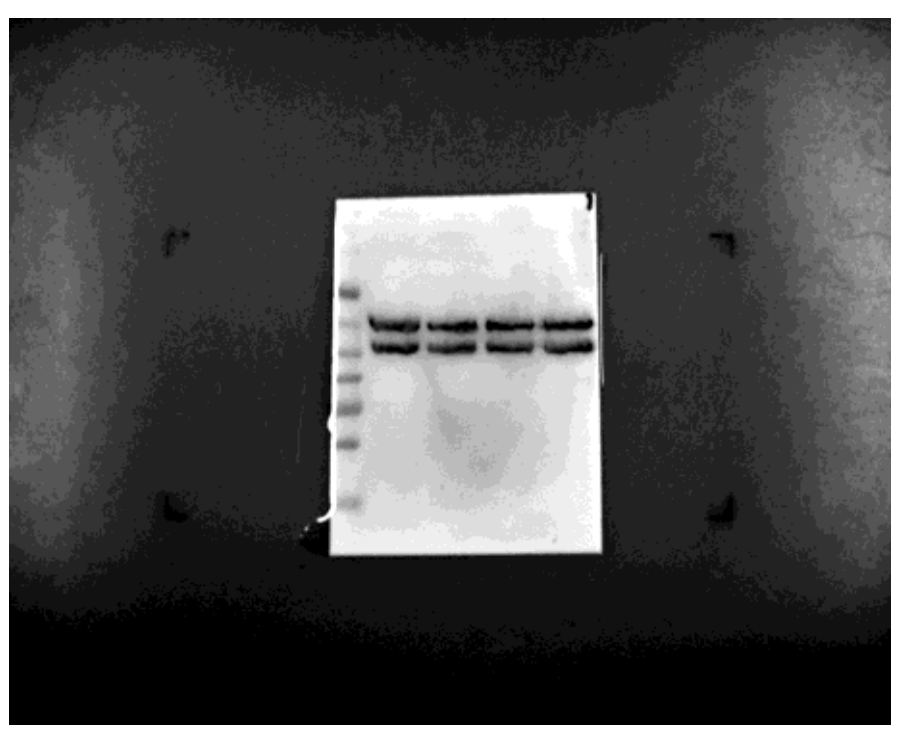

JNK

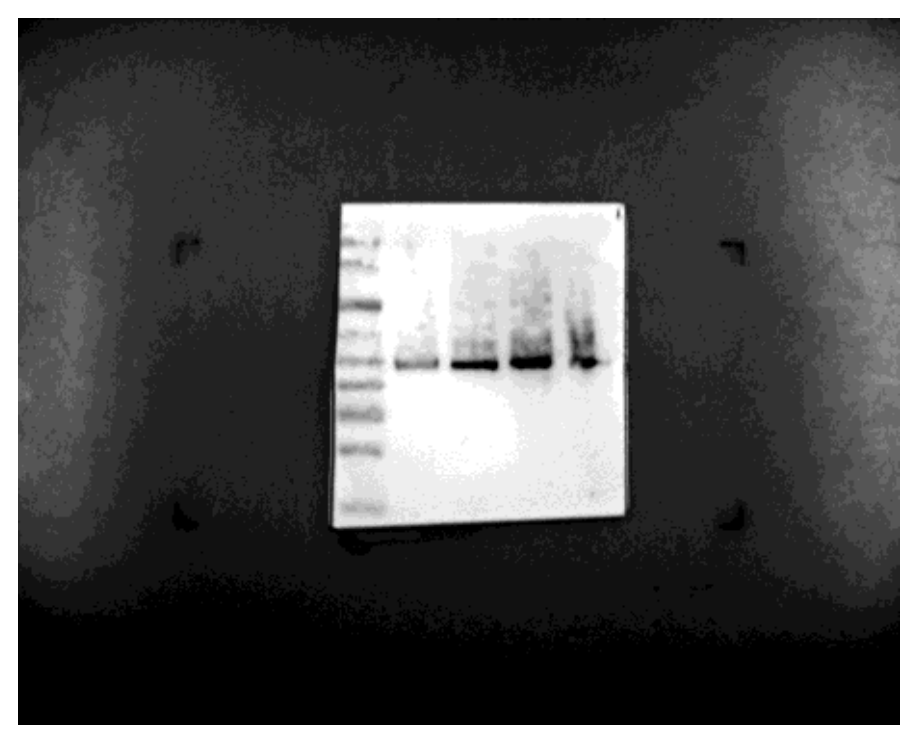

p-p38

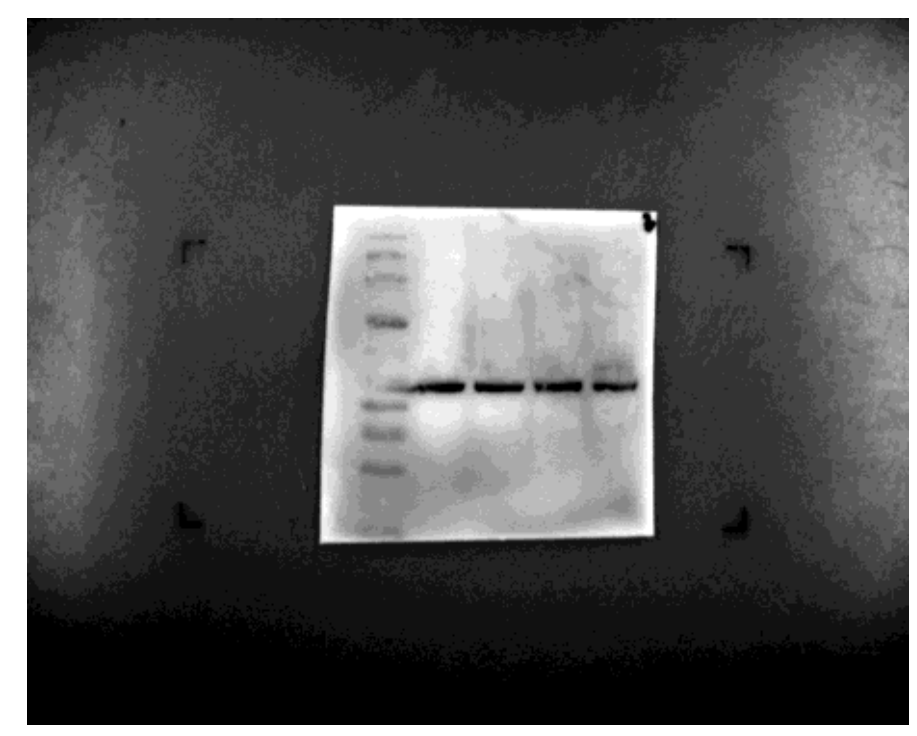

p38

Western blot

Figure 8

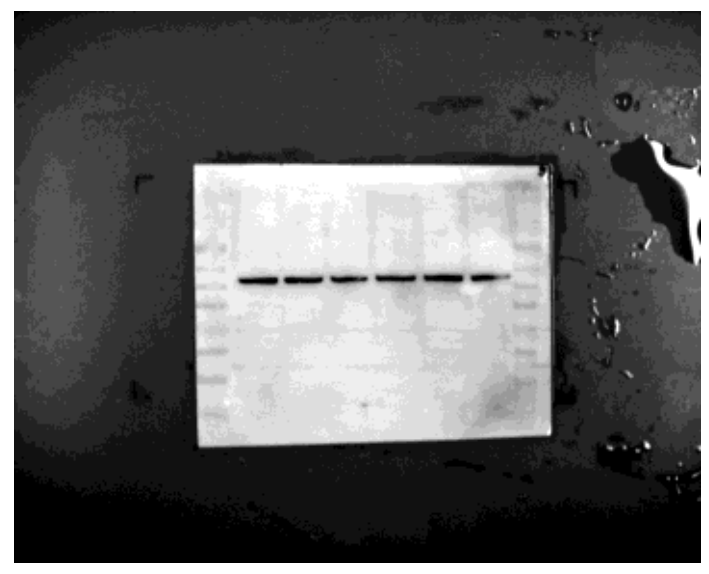

β-actin

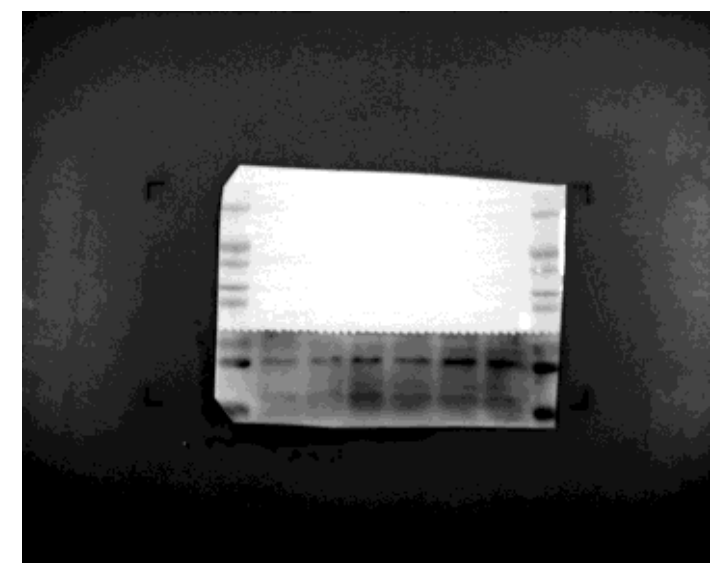

MSC

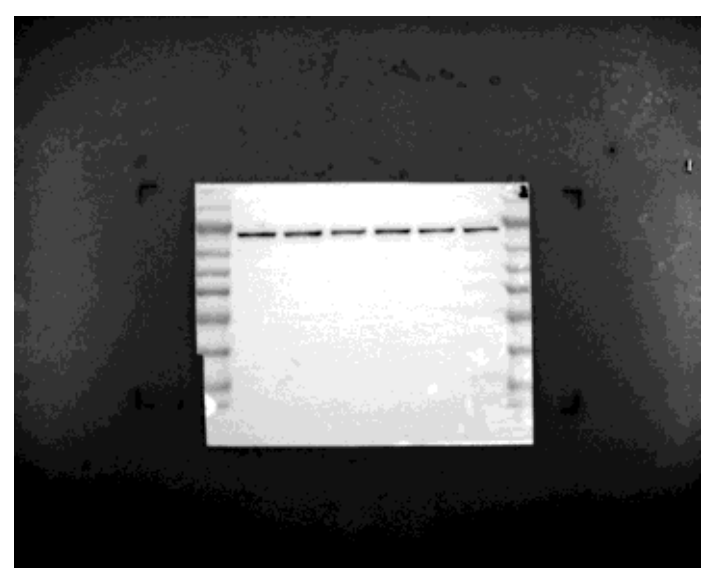

p65

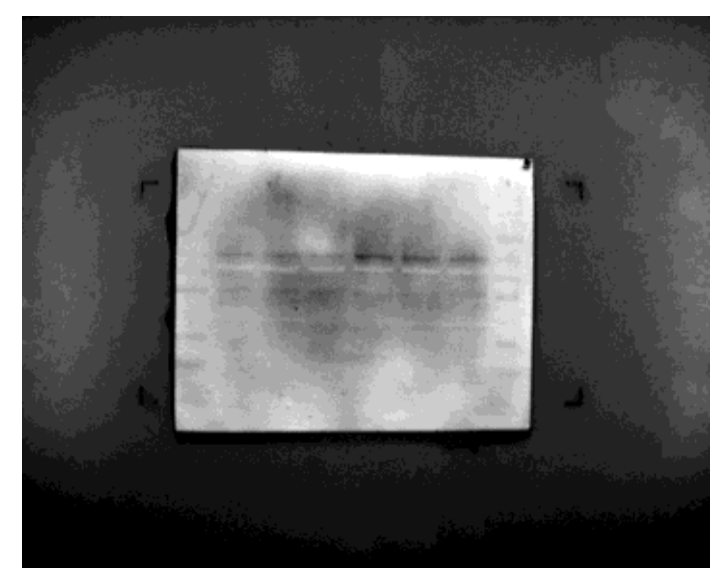

p-p65

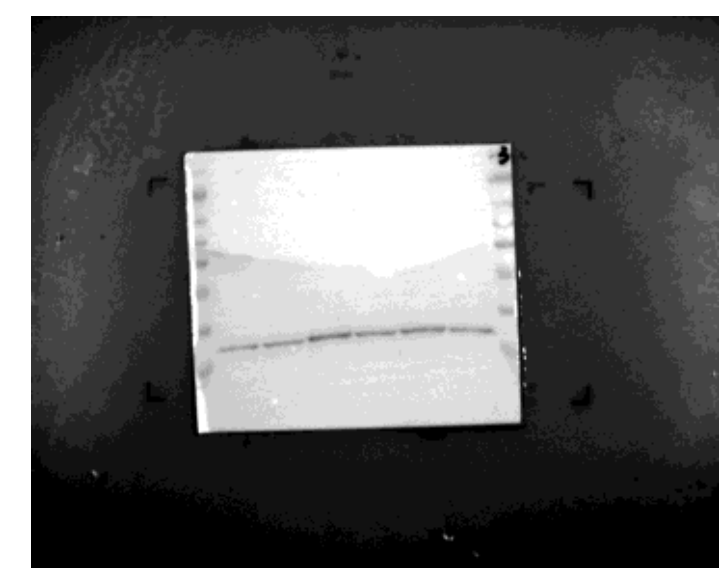

H3

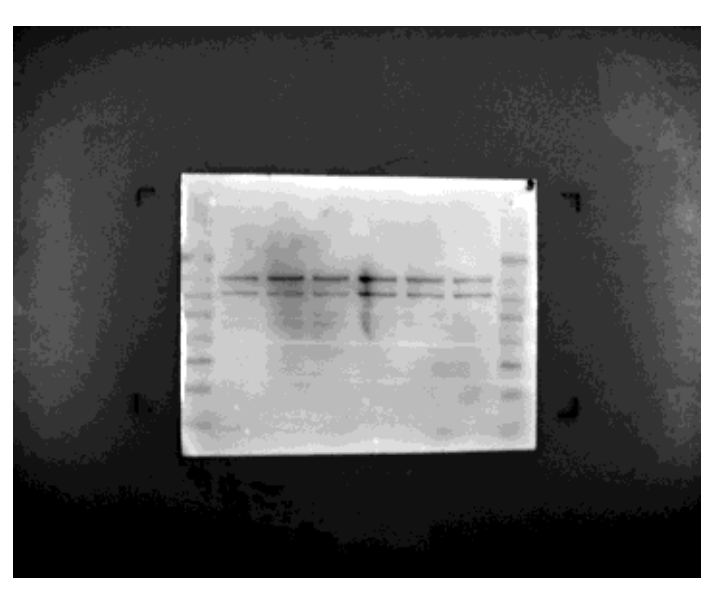

JNK

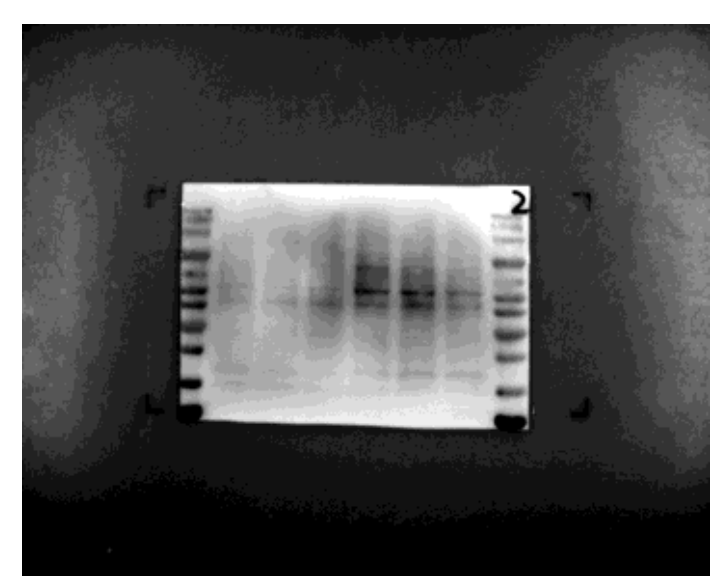

p-JNK

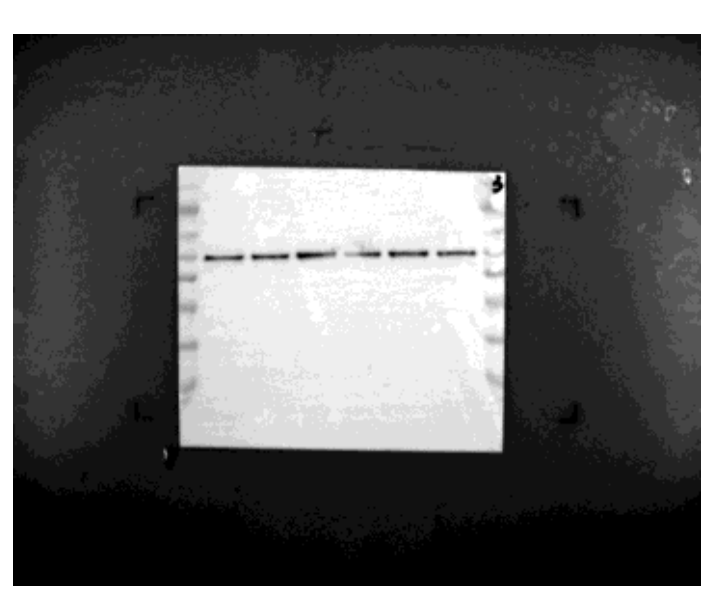

p38

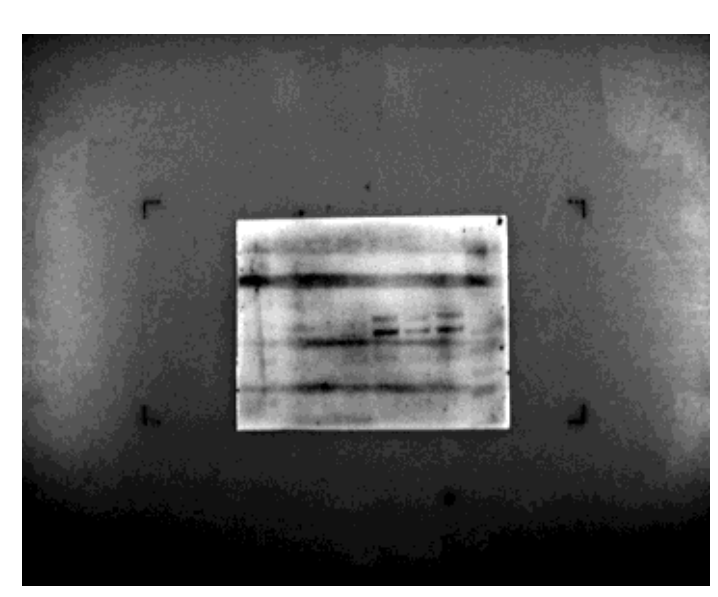

p-p38

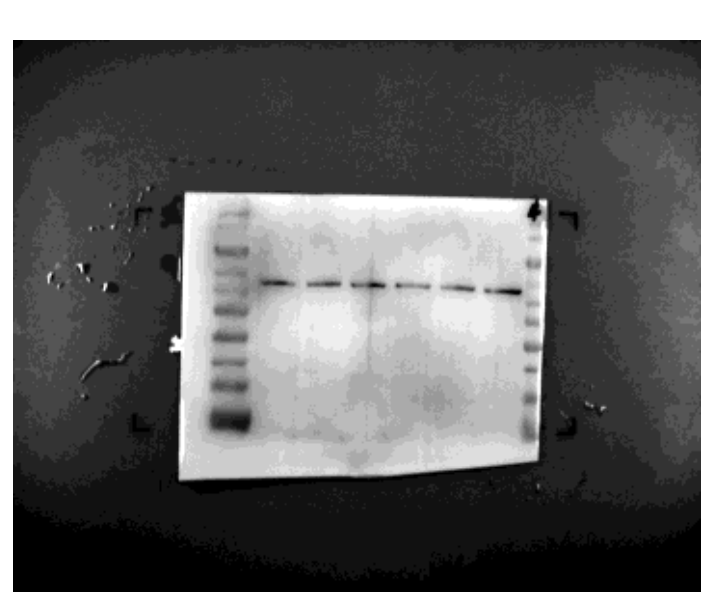

β-actin

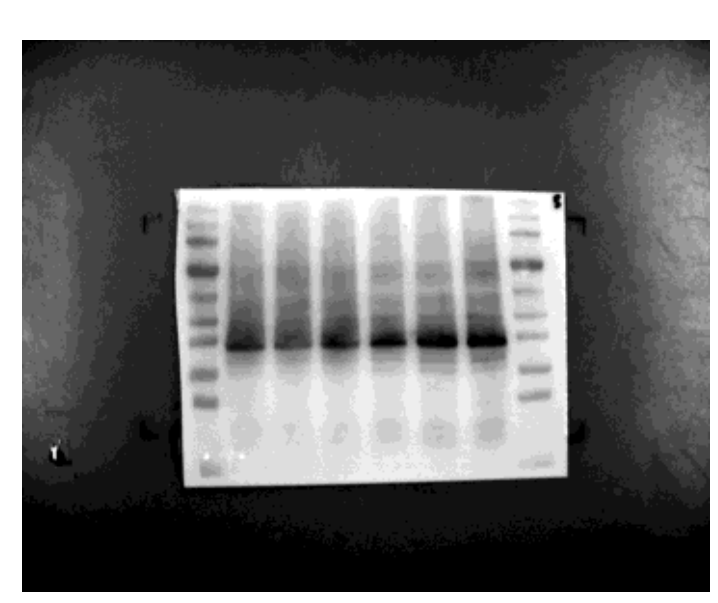

METTL1
