# Supplementary material for: Methyltransferase METTL1 regulates MSC mRNA stability via m7G modification in acute pancreatitis
Source: Cell Death Dis. 2026 Jun 23;17(1):775. doi: 10.1038/s41419-026-09002-7 (PMC13538475; doi:10.1038/s41419-026-09002-7)
Supplement: Supplementary file 3 [file 41419_2026_9002_MOESM1_ESM.docx]

**Supplementary Informations**

**Methyltransferase METTL1 regulates MSC mRNA stability via m7G modification in acute pancreatitis**

Xufeng Tao^1^*, Xiaonan Zhang^2,3^*, Fangyue Guo^2^, Yunshu Zhang^2,3^, Lei Zhang^1,4^, Han Yu^2,3^, Deshi Dong^1^, Dong Shang^2,3,5^, Hong Xiang^2#^

^1^Department of Pharmacy, First Affiliated Hospital of Dalian Medical University, Dalian, 116011, China.

^2^Laboratory of Integrative Medicine, First Affiliated Hospital of Dalian Medical University, Dalian, 116011, China.

^3^Institute (College) of Integrative Medicine, Dalian Medical University, Dalian, 116044, China.

^4^Department of Pharmacy, Dalian Medical University, Dalian, 116044, China.

^5^Department of General Surgery, First Affiliated Hospital of Dalian Medical University, Dalian, 116011, China.

*These authors contributed equally to this work.

^#^Corresponding author: Hong Xiang, Laboratory of Integrative Medicine, First Affiliated Hospital of Dalian Medical University, Dalian, 116011, China.

E-mail: xianghong@dmu.edu.cn

Materials and Methods

AP mouse model

Male C57BL/6J mice (22 - 25 g, 6 - 8 weeks old) were purchased from HFK Bioscience Co., Ltd (Beijing, China). Mice were randomly allocated to two groups (n = 5 per group): control (Ctrl) and acute pancreatitis (AP). The sample size was selected based on previous studies using the sodium taurocholate–induced AP model and our pilot experiments, without a priori statistical power calculation.

AP was induced as follows description. Briefly, following anesthesia induction with isoflurane inhalation, a midline abdominal incision was made to expose the pancreas. Acute pancreatitis was established by retrograde injection of 5.0% sodium taurocholate (STC; Solarbio, Beijing, China) into the pancreaticobiliary duct (0.1 mL/100 g body weight). Control mice received an equivalent volume of physiological saline via the same procedure.

All mice were euthanized 24 h after surgery, and serum and pancreatic tissues were collected for subsequent analyses. Animals were excluded only if pre-established criteria were met (e.g., technical failure of duct cannulation/injection, accidental death unrelated to the experimental endpoint, or insufficient sample for downstream assays); otherwise, no animals were excluded from analysis. Outcome assessment and data analysis were performed in a blinded manner.

**Enzymatic detection**

The serum amylase and lipase were detected using Commercial Detection Kit purchased from Nanjing Jiancheng Bioengineering Institute (Nanjing, China), according to the manufacturer's instructions. The absorbance at 660 nm was measured under a microplate reader (BioTek; CA, USA).

Hematoxylin and eosin (H&E) staining

Tissue samples fixed with 4% paraformaldehyde were embedded in paraffin. Paraffin sections (5 μm) of the pancreas were dewaxed with xylene solution, hydrated with gradient alcohol, stained with hematoxylin and eosin (H&E), and photographed under an optical microscope (Leica DM4000B; Wetzlar, Germany) at 200× magnification. Pancreatic pathology scoring referenced to the previous standards.

Immunohistochemistry (IHC) and Immunofluorescence (IF)

Tissue sections were dewaxed, hydrated, and antigen retrieved, followed by incubation with METTL1 (1:50; 14994-1-AP; Proteintech; Hubei, China), m7G (1:2000; 68302-1-Ig; Proteintech; Hubei, China), F4-80 (1:100; 123116; BioLegend; CA, U.S.A), iNOS (1:100; sc-7271; Santa Cruz Biotechnology; Texas, U.S.A), AMS (1:100; sc-46657; SantaCruz Biotechnology; Texas, U.S.A), CK19 (1:100; 628504; BioLegend; CA, U.S.A) antibodies overnight at 4 °C. The sections then were incubated with suitable secondary antibodies at room temperature for 1 h. Images were obtained using an optical microscope (Olympus; Tokyo, Japan), and positive staining areas were quantified using ImageJ software (1.53t with 64-bit Java 8).

**Clinical sample collection**

A cohort of 70 subjects was enrolled at the First Affiliated Hospital of Dalian Medical University, including 35 patients diagnosed with acute pancreatitis (AP) and 35 age- and sex-matched healthy adults. The sample size was determined based on feasibility and availability of eligible participants during the study period and was consistent with prior similar studies; no a priori statistical power calculation was performed.

For the AP group, patients with severe chronic underlying diseases, malignant tumors, or gastrointestinal diseases were excluded according to pre-established criteria. For the control group, healthy volunteers were recruited with age and gender matching and without chronic metabolic diseases, gastrointestinal diseases, tumors, or other major diseases, based on pre-established eligibility criteria. Demographic characteristics and clinical indicators were summarized in Supplementary Tables S1–S2. Blood samples were collected after admission (AP group) or at recruitment (controls) and centrifuged to obtain serum. Serum samples from all subjects were analyzed by ELISA. Laboratory measurements and data analysis were performed in a blinded manner with respect to group allocation.

Cell culture

Mouse macrophages RAW264.7 cells (ATCC TIB-71), human embryonic kidney cells HEK-293T (ATCC CRL-11268), and mouse PACs 266-6 cells (ATCC CRL-2151) were purchased from American Type Culture Collection (ATCC, Manassas, VA, USA). Cells were separately cultured in DMEM medium containing 10% fetal bovine serum in a humidified 37 °C incubator with 5% CO_2_. During our use, it has been tested that there has never been mycoplasma contamination.

Cell viability

The 266-6 cells were incubated with STC solution at increasing concentrations (0, 100, 200, 500, 600, 700, 800, 900, 1,000, 1,500 μM) for 24 h, and then incubated with 10 μL of 0.4 mg/mL MTT solution at 37 °C for 4 h. After discarding the supernatant, the formazan crystals were dissolved with 100 μL DMSO. The absorbance at 490 nm was measured using a microplate reader (BioTek; CA, USA).

Flow cytometry

RAW264.7 cells were permeabilized with fixation and permeabilization buffer (BD Biosciences; NJ, USA) at 4 °C, stained with F4/80 (1:100; sc-377009; Santa Cruz Biotechnology; TX, USA) and iNOS (1:100; c-7271; Santa Cruz Biotechnology; TX, USA) antibodies, and detected by LSRFortessa™ Cell analyzer (BD Biosciences; NJ, USA).

Dot blot

RNA sample was dropped on the NC membrane that was cross-linked with ultraviolet light at a wavelength of 254 nm, and orderly incubated with m7G antibody (1:7000; 68302-1-Ig; Proteintech; Hubei, China) overnight at 4 °C, following by the secondary antibodies at room temperature for 1 h, and with enhanced chemiluminescence solution (ECL) (Tanon 4200; Shanghai, China) for 5 min. The membrane was stained with 0.02% methylene blue staining buffer and photographed under a developer. White light images were collected as a loading control. Total RNA was accurately quantified to ensure equal loading across samples prior to dot blotting, and methylene blue staining served as the loading control.

Plasmid transfection

HEK293T cells and RAW264.7 cells were seeded in 6-well plates for 24 h and transfected with *Mettl1* over-expression (oe-*Mettl1*) plasmids or *Mettl1* interference (sh-*Mettl1*) plasmids (GenePharma; Shanghai, China). Cells transfected with an empty vector served as negative controls (NC). After 24 h of transfection, cells were subjected to additional treatments as described in this study. METTL1 catalytic-dead mutant was constructed based on the study by Lin *et al*^.^, where the amino acid sequence at positions 160-163 (LFPD) was mutated to AFPA (Hengyuan Biotechnology; Shanghai, China).

m7G-MeRIP sequencing

m7G-MeRIP sequencing was entrusted to Cloud-Seq Biotech Ltd. Co. (Shanghai, China) for assistance. Briefly, m7G-IP reaction was performed using the RNA acetylation kit from GenSeq (Shanghai, China). The RNA was randomly fragmented into fragments of approximately 200 nt. After the protein A/G magnetic beads were incubated with the m7G antibody at room temperature for 1 h, the RNA fragments were incubated with the antibody bound to the magnetic beads at 4 °C for 4 h. The RNA sequencing library was constructed using the GenSeq^®^ Low Input Whole RNA Library Prep Kit (GenSeq, Inc.). The Agilent 2100 Bioanalyzer was used for quality control, and the raw reads were generated after high-throughput sequencing, image analysis, base recognition, and quality control on the Illumina NovaSeq sequencer. Then, cutadapt software (v1.9.3) was used to remove the adapters and low-quality reads to obtain high-quality clean reads. The clean reads of all samples were matched to the reference genome using Hisat2 software (v2.0.4). The methylated mRNAs in each sample were identified using MACS software. Differentially methylated mRNAs were identified using diffReps software. Peaks located on the exons of mRNA were screened using a proprietary program and annotated accordingly.

m7G-MeRIP qPCR

Differentially methylated mRNAs identified through m7G-MeRIP-seq were validated via qPCR. RNA immunoprecipitation was performed with GenSeq^®^ m7G MeRIP kit (GS-ET-004; Cloud-Seq; Shanghai, China) according to the instructions. Small amounts of fragmented RNA were used as starting controls, while the remaining RNA was incubated with magnetic beads conjugated with m7G or IgG antibodies. Immunoprecipitated complexes eluted from the beads were subjected to qPCR for quantitative analysis. Primer sequences were listed in **Table S3**.

RNA sequencing

RAW264.7 cells were transfected with lentiviral overexpression vector carrying the Musculin (MSC) gene (Hanheng Biotech Co., Ltd; Shanghai, China) at an appropriate multiplicity of infection (MOI = 20) for 24 h. Total RNA was extracted using TRIzol reagent (Accurate Biology; Hunan, China) according to the manufacturer's instructions. Then, mRNA was isolated using Oligo(dT)-containing magnetic beads with ployA for A-T base pairing, and fragmented using fragmentation buffer. The cDNA was reverse synthesized, and the adapter sequence was added. The final library was obtained by purification, fragment sorting, and re-amplification, which was sequenced. Library preparation and sequencing were performed by Majorbio Bio-Pharm Technology Co., Ltd (Shanghai, China).

qPCR

Total RNA was extracted using the RNAex Pro RNA reagent (Accurate Biology; Hunan, China) following the manufacturer’s instructions. RNA was reverse transcribed using the Evo M-MLV RT MIX Kit (Accurate Biology; Hunan, China), and relatively quantified by SYBR^®^ Green Premix Pro Taq HS qPCR Kit (Accurate Biology; Hunan, China) in the ABI 7500 qPCR system (Applied Biosystems; California, USA). Primer sequences were listed in **Table S4**. All gene expression levels were normalized to β-actin.

Western blot

Protein extracts were separated by SDS-PAGE and transferred to polyvinylidene fluoride (PVDF) membranes (Millipore; MA, USA). Membranes were blocked with 5% nonfat milk or 5% BSA for 1 h at room temperature, incubated overnight at 4 °C with primary antibodies against METTL1 (1:5000; 14994-1-AP; Proteintech; Wuhan, China), histone H3 (acetylK14) (1:2000; ab52946; Abcam; Cambridge, UK), MSC (1:750; abs148045; Absin; Shanghai, China), nuclear factor-κB (NF-κB) p65 (1:1000; 8242S; CST; MA, USA), phospho-NF-κB p65 (1:1000; 3033S; CST; MA, USA), c-Jun N-terminal kinase (JNK) (1:1000; 9252S; CST; MA, USA), phospho-JNK (1:1000; 60666-1-Ig; Proteintech; Wuhan, China), mitogen-activated protein kinase (MAPK) p38 (1:1000; 9212S; CST; MA, USA), phospho-MAPK p38 (1:1000; 4511S; CST; MA, USA), β-actin (1:20000; AC043; Abclonal; Wuhan, China), and then incubated with HRP-conjugated Goat anti-Rabbit IgG (H+L) (1:2000; AS014; Abclonal; Wuhan, China) for 1 h at room temperature. Signals were detected using a chemiluminescence detection system (Tanon 5200; Shanghai, China).

**Conditioned medium preparation and co-culture**

RAW264.7 cells were seeded in 6-well plates and cultured for 24 h. After adherence, the cells were treated with either LPS (1 µg/mL) or LPS combined with sh-*Mettl1* plasmids. After 24 h of treatment, the conditioned medium was collected by gently aspirating the supernatant from each well, ensuring no cell contamination. The collected supernatant was filtered through a 0.22 µm sterile filter to remove cell debris. Simultaneously, the 266-6 cells were seeded in separate 6-well plates and treated with the conditioned medium collected from RAW264.7 cells. All treatments were maintained for 24 h, after which further analysis was performed as per the experimental protocol.

**AAV9-mediated *Mettl1* knockdown in mice**

To achieve *Mettl1* knockdown *in vivo*, AAV9 serotype viral vectors were administered via tail vein injection. C57BL/6 mice weighing 20-30 g were injected with AAV9 virus at a titer of 10^13 GC/mL, containing GFP fluorescence and driven by the U6 promoter. Each mouse received an injection of 150 μL of viral solution at a rate of 6 μL/min. After 6 weeks of injection, tissue collection was performed. The pancreas was harvested for histopathological analysis and immunohistochemical staining, while part of the pancreas tissue was used for qPCR validation of the *Mettl1* knockdown efficiency. Additionally, serum samples were collected for subsequent enzymatic assays.

**Statistical analysis**

Data are presented as mean ± standard error of the mean (SEM). Statistical analyses were performed using GraphPad Prism 9.0 (GraphPad Software, La Jolla, CA, USA). A two-tailed Student’s t-test was used to evaluate differences between two groups. One- or two-way ANOVA was used to evaluate the statistical significance among multiple groups. The exact sample size (n) and the statistical test used are indicated in the figure legends; n represents the number of independent biological replicates (individual mice or human subjects, as applicable). *P* value < 0.05 was considered statistically significant.

**Supplementary Figure S1. Construction and validation of the catalytic-dead METTL1 mutant.** (A) Sequence alignment of METTL1 highlighting the conserved catalytic motif **LFPD** (aa160–163) between human and mouse, which was mutated to **AFPA** (B) **for** a catalytic-dead METTL1 mutant. (C) Representative bright-field and GFP fluorescence images showing expression of the Mut-GFP construct in macrophages (40×). Scale bar, 250 μm.

Table S1. Baseline characteristics of participants in the clinical trial.

| Characteristics | Ctrl (n=35) | AP (n=35) | p-value |
| --- | --- | --- | --- |
| Age (year) | 56.09±10.38 | 54.37±12.58 | 0.536149 |
| Gender | 45.71% (Male) | 54.29% (Male) | 0.624206 |

Table S2. Analysis of the levels of relevant clinical indicators in AP patients.

| Characteristic | AMY (U/L) | LPS (U/L) | ALT (U/L) |
| --- | --- | --- | --- |
| AP (n=35) | 969.83±1167.92 | 8232.18±10277.54 | 79.23±113.74 |
| Characteristic | **AST (U/L)** | **ALP (U/L)** | **γ-GT (U/L)** |
| AP | 56.46±79.31 | 115.91±90.49 | 172.57±201.83 |
| Characteristic | **T-BIL (μM)** | **DBIL (μM)** | **A/G** |
| AP | 37.22±43.56 | 17.26±29.52 | 2.87±10.14 |
| Characteristic | **GLU (mM)** | **Urea (mM)** | **CRE (μM)** |
| AP | 9.62±4.35 | 8.02±16.84 | 78.83±68.94 |

Table S3. Primer sequences used for MeRIP-qPCR assay.

| Gene | Species | Primers (5'-3') |
| --- | --- | --- |
| *MSC* | human | Forward: CGCTTCCTCTTGCAGCCTTCC  Reverse: CAGTGACAACTCGTCGGCAGAG |
| *GPR37L1* | human | Forward: CACGCCCAGCCCCAGTAC  Reverse: AAAGAAAGGAGGAGGAAGGAGAGAG |
| *HSFX2-1* | human | Forward: GCCAGATGGAGGACAAGCGATC  Reverse: GCACCCTTTCCAAGCCATGATCC |
| *HSFX2-2* | human | Forward: AGCCAGGCTCACGTCACTCC  Reverse: ACTACGGGACGCTGTGGGAAG |
| *CCR10* | human | Forward: TGGCAAGGCACAGAGGTAGTCC  Reverse: AGGCTGAGGGTCGTGGGAAAG |
| *DOK7* | human | Forward: TCCTTGAGAGCCGCAGATCCC  Reverse: CCACAGAACACAGAGCACCAGTC |

Table S4. Primer sequences used for qPCR assay.

| Gene | Species | Primers (5'-3') |
| --- | --- | --- |
| *β-Actin* | mouse | Forward: ACTGCCGCATCCTCTTCCTC  Reverse: AACCGCTCGTTGCCAATAGTG |
| *Cxcl1* | mouse | Forward: TGGCTGGGATTCACCTCAAGAAC  Reverse: TGTGGCTATGACTTCGGTTTGGG |
| *Cxcl2* | mouse | Forward: CCACCAACCACCAGGCTACAG  Reverse: GGGCGTCACACTCAAGCTCTG |
| *Cxcl5* | mouse | Forward: TTCCTCAGTCATAGCCGCAACG  Reverse: GACTTCCACCGTAGGGCACTG |
| *Il-6* | mouse | Forward: GAGAGGAGACTTCACAGAGGATACC  Reverse: TCATTTCCACGATTTCCCAGAGAAC |
| *Il-8* | mouse | Forward: TGATGCCACCGCCTCCTCTG  Reverse: AAAGGTTTCCTGACTGACCGAACG |
| *Mettl1* | mouse | Forward: CTGTGGCTATGGTGGCTTGTTAG  Reverse: GCACATAGTCCGACACCTTCAC |
| *Wdr4* | mouse | Forward: TTTGTGAGCCGCATCCTTGTAGTG  Reverse: CCAGAGTCTCAGGGTGCCATCC |
| *Tnf-α* | mouse | Forward: ACGTGGAACTGGCAGAAGAGG  Reverse: TGAGAAGAGGCTGAGACATAGGC |
| *Il-1β* | mouse | Forward: TCGCAGCAGCACATCAACAAGAG  Reverse: AGGTCCACGGGAAAGACACAGG |
| *Inos* | mouse | Forward: CAACTACTGCTGGTGGTGACAAG  Reverse: CTGAAGGTGTGGTTGAGTTCTCTAAG |
| *Cd86* | mouse | Forward: AGCACTATTTGGGCACAGAGAAAC  Reverse: GTGAAGTCGTAGAGTCCAGTTGTTC |
| *Cxcl13* | mouse | Forward: GTGTGAATCCTCGTGCCAAATGG  Reverse: CTGGAGCTTGGGGAGTTGAAGAC |
| *Ccl17* | mouse | Forward: CCGAGAGTGCTGCCTGGATTAC  Reverse: TGCCCTGGACAGTCAGAAACAC |
| *Msc* | mouse | Forward: CTGAGCAAAGCCTTCTCCAGACTG  Reverse: GTCCTCGTAGCGGTCCTCCTG |
| *Fos* | mouse | Forward: ACCCTGAGCCCAAGCCATCC  Reverse: CGGGAGGTCTCTGAGCCACTG |
| *Ptgs2* | mouse | Forward: GGTGCCTGGTCTGATGATGTATGC  Reverse: GGATGCTCCTGCTTGAGTATGTCG |
| *Lif* | mouse | Forward: GCAACGGGACAGAGAAGACCAAG  Reverse: ACGGCAGTGGGGTTCAGGAC |
| *Cxcl10* | mouse | Forward: TGCCTCATCCTGCTGGGTCTG  Reverse: TCCCTATGGCCCTCATTCTCACTG |
| *Ccl5* | mouse | Forward: GACACCACTCCCTGCTGCTTTG  Reverse: CTCTGGGTTGGCACACACTTGG |
| *Ccl2* | mouse | Forward: CCACTCACCTGCTGCTACTCATTC  Reverse: CTCCCAGCCAGGTGTCATTTTC |
| *Ccl3* | mouse | Forward: AGTTCCAGGTCAGTGATGTATTCTTG  Reverse: GATGACGATGATGATGACGATGGAG |
| *Spp1* | mouse | Forward: TGTGTACTAGCAGTGACGGTCTC  Reverse: GATCCCAGAAGACGAATAGGTT |
| *CCR10* | human | Forward: CACACTTGGTCTCCGTCATC  Reverse: GTAGCAGGCTACCATGACG |
| *ART1* | human | Forward: ATGTCTCTGCTTCTTGTGTCTGTGG  Reverse: GCCTGGAACTCCGTGTGGTTG |
| *APC2* | human | Forward: CTGAAGCACCTACAGGGAAAA  Reverse: CTGGAACTTGAGGTTGTACAGG |
| *ADCY2* | human | Forward: TCATCACCACAGCCATCATATT  Reverse: GGAAAAATAAATTCTGCGCACG |
| *GALNT5* | human | Forward: TAATGTGGCTTTGGGTAAATGC  Reverse: GGCTATACACCATTCTCCACAT |
| *USHBP1* | human | Forward: GCAGGACTGACAAGAAGATGGATGG  Reverse: GCTGGAGGGTCTGAAACACATCG |
| *TRPA1* | human | Forward: GCACAGATCGCCTGGATGAAGAC  Reverse: TGTCAGCATTGTGGCTCAGAAGAAG |
| *THSD7A* | human | Forward: CTTCCAATGACCTTCCAGTCCTGTG  Reverse: CTGCCTGATGGTTCGTGTCCTTAC |
| *SYNPO2* | human | Forward: GTTACCAGACACCACAGGCAAGG  Reverse: GCTCTGCGTTCTTACCGACTCTTC |
| *STMND1* | human | Forward: AAGCCATTGAGAAAGCCACCTTCC  Reverse: TCCTCGGCAGCCTCCATCTTC |
| *SRPK3* | human | Forward: CTATTCCCGGGAGTTCTTCAAC  Reverse: TTTCCATGAGTACCTCGTACAG |
| *SLC22A31* | human | Forward: AAACGGGCTTATCTTGGGCTTCAG  Reverse: CCAGGAAGTAGGGCAGGTAGAAGG |
| *RNF148* | human | Forward: GTGTCTGGTGTGGTGGCACTTC  Reverse: CCCATTTGCTCCCTTCTCTGCTG |
| *PTN* | human | Forward: CTGCAACTGGAAGAAGCAATTT  Reverse: GTCCAATACCTCACTCGCTCAGC |
| *PLN* | human | Forward: GGACTTCATTTCCTACACTCCC  Reverse: TTTGACGTGCTTGTTGAGGCATTTC |
| *PLIN5* | human | Forward: CCTGGCTGAGAACTGCGTGTG  Reverse: CGAAGGTTGCTGGAGAAAGGGAAG |
| *MSC* | human | Forward: CTTCCAGTTACATCGCTCACC  Reverse: GGAAACTTCTTTGGTGTCAGAG |
| *MIP* | human | Forward: TCTCACTGGGAACTTCACTAAC  Reverse: GGGAAGAGAAGAAAGTCGTACA |
| *JMJD7-PLA2G4B* | human | Forward: CTTCATCCCCTATGAGCTGTAC  Reverse: GTCACTAGGTCCTTAGAGGGTA |
| *HSFX2* | human | Forward: ACCAAGACATCAGGAGGAGGACAAG  Reverse: GGCGGAATCAGGCACCATCAC |
| *GPR37L1* | human | Forward: CATGCATCATGAAACCCTCAG  Reverse: AGAAGTAGCAGCCAAAGTACC |
| *DOK7* | human | Forward: AGTGGAAGAGTAGGTGGCTGGTG  Reverse: GATACGCTCCGACTTGTCCTTGTAG |
| *CHGB* | human | Forward: AGAAAGCAGGCTTCAGCTATAA  Reverse: CTACTCTTCTCTCGGCTATGTG |
| *CDH4* | human | Forward: GAGACTGGAGATATCGTCACAG  Reverse: GTTGTCATTCACATCTGTCACC |
| *WNK4* | human | Forward: GGACTTCATTTCCTACACTCCC  Reverse: GTAGGTCCCGTGATAAAGACAT |
